# Signaling Logic of Activity-Triggered Dendritic Protein Synthesis: An mTOR Gate But Not a Feedback Switch

**DOI:** 10.1371/journal.pcbi.1000287

**Published:** 2009-02-13

**Authors:** Pragati Jain, Upinder S. Bhalla

**Affiliations:** National Centre for Biological Sciences, Tata Institute of Fundamental Research, Bangalore, India; University College London, United Kingdom

## Abstract

Changes in synaptic efficacy are believed to form the cellular basis for memory. Protein synthesis in dendrites is needed to consolidate long-term synaptic changes. Many signals converge to regulate dendritic protein synthesis, including synaptic and cellular activity, and growth factors. The coordination of these multiple inputs is especially intriguing because the synthetic and control pathways themselves are among the synthesized proteins. We have modeled this system to study its molecular logic and to understand how runaway feedback is avoided. We show that growth factors such as brain-derived neurotrophic factor (BDNF) gate activity-triggered protein synthesis via mammalian target of rapamycin (mTOR). We also show that bistability is unlikely to arise from the major protein synthesis pathways in our model, even though these include several positive feedback loops. We propose that these gating and stability properties may serve to suppress runaway activation of the pathway, while preserving the key role of responsiveness to multiple sources of input.

## Author Summary

Memory formation involves the controlled production of new proteins close to the site of input stimuli on nerve cells. Strong inputs, in combination with growth factors, stimulate the synthesis of several kinds of synaptic proteins. These new proteins are believed to participate in remodeling the contacts between cells. This gives rise to a potentially unstable situation of a self-modifying cellular machine, because the new proteins rebuild their own inputs and their own production machinery. We have analyzed these interactions by modeling multiple inputs and the process of self-modifying feedback. We find that runaway modifications are prevented in two ways: first, a molecule called mTOR acts as a gate to suppress synthesis except under very tightly regulated conditions. Second, the feedback processes operate in a range where it is very unlikely that they can give rise to runaway buildup. Thus, the system avoids instability even though it is capable of modifying itself in response to many kinds of inputs.

## Introduction

Protein synthesis is a necessary stage in long-term synaptic plasticity, both for strengthening synapses (long-term potentiation, LTP) and weakening them (long-term depression, LTD) [1],[2]. A substantial part of this synthesis occurs in the dendrites, and possibly close to the modified synapse(s) [3]. mRNAs are transported to the dendrites where local sites of protein synthesis machinery appear to serve a small number of synapses or dendritic spines [4]. There are now several putative signaling pathways that connect synaptic input to protein synthesis [5],[6]. The relationship between synthesis triggered by activity, different receptors, and growth factors is important in defining the logic of memory formation [7],[8].

Dendritic protein synthesis is a particularly obvious example of a positive feedback process, since many of the newly synthesized proteins either provide signal input to the system, or are part of the synthesis machinery itself. From the viewpoint of memory formation, positive feedback is a significant feature: it suggests that self-sustaining activation processes may occur [9]. Such self-sustaining processes frequently take the form of bistable switches, and indeed a protein synthesis switch has been proposed to sustain memory in Aplysia [10]. Many biochemical and signaling switches have been proposed at the mammalian synapse, for example, calcium calmodulin type II kinase (CaMKII) autophosphorylation [11], MAPK-PKC feedback [12],[13], and receptor cycling [14]. Given its known role in synaptic plasticity, it is tempting to consider the possibility that protein synthesis may also form such a switch.

**Figure 1:**
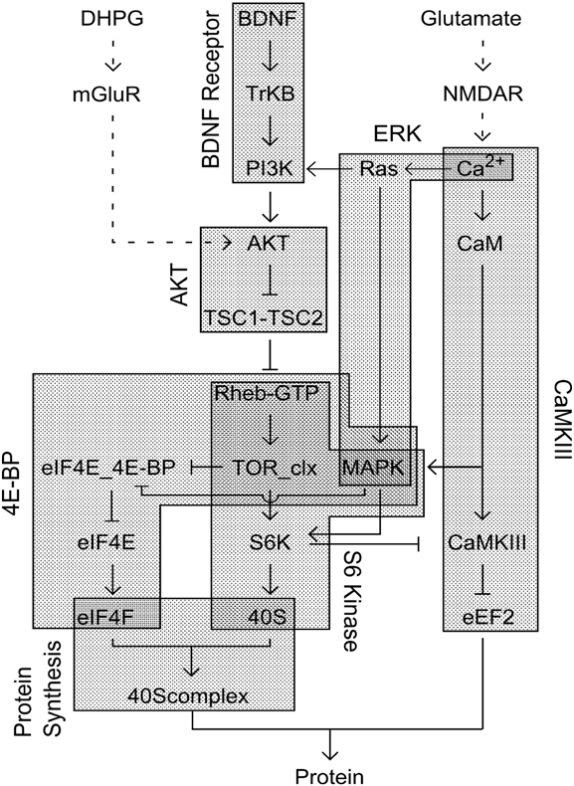
Block diagram of signaling network. Inputs are located at the top. The primary input to the pathway is BDNF. We use Ca^2+^ influx as a surrogate for glutamate input, and AKT activation as equivalent to input from the mGluR5 pathway, since it is still incompletely understood. Dashed arrows represent such virtual inputs. Regular arrows represent positive interactions and T-s represent inhibitory inputs. The various modules in the model are represented as gray blocks, and their names are in rotated text.

Synaptic protein synthesis has attracted considerable recent attention, and several of its key pathways have been identified. Major inputs include neurotrophins such as BDNF [15] and neurotransmitters such as glutamate, which in turn may act through metabotropic [16] and ionotropic [17] pathways. The BDNF pathway has been extensively studied and at least one rather lengthy signaling cascade has been identified that culminates with protein synthesis regulation (Figure 1). In this cascade, BDNF binds to its receptor, tropomyosin-related kinase B (TrkB), which activates phosphatidylinositol-3-kinase (PI3K), then AKT, which elevates Rheb-GTP to activate the mammalian target of rapamycin (TOR). TOR acts on synthesis both through its phosphorylation of the S6 Kinase (S6K), and through its effects on eukaryotic initiation factor 4E (eIF4E). Both of these are involved in translation initiation and hence control protein synthesis. The metabotropic glutamate receptor (mGluR) regulation of synthesis is less understood, but there is evidence that it joins this same cascade at AKT [18], though the upstream steps are yet to be resolved. Ionotropic-glutamate signaling works through the association of postsynaptic depolarization and presynaptic glutamate, leading to opening of the N-methyl-d-aspartate receptor (NMDAR) and calcium (Ca^2+^) influx. Calcium has multiple effects on protein synthesis. Two of the possible inputs are via Mitogen-activated protein kinase (MAPK, present in neurons as Extracellular signal-regulated kinase II, ERKII) and via calcium-calmodulin type III kinase (CaMKIII). MAPK, like mTOR acts on S6Kinase and 4E-binding protein (4EBP) which blocks eIF4E. CaMKIII phosphorylates eukaryotic elongation factor-2 (eEF2) and inhibiting its elongation activity (Figure 1).

These downstream targets (eIF4E and 40S) represent possible control points for selective protein synthesis. This is because 40S phosphorylation has been particularly implicated in the translation of a subset of mRNAs with a 5′ terminal oligopyrimidine sequence (5′TOP). 5′TOP proteins are involved in protein synthesis, and the 40S (S6, a subunit of 40S) protein itself has a 5′TOP [5]. In the absence of such 5′TOP targeting, other dendritic mRNAs are translated, including Arc, αCaMKII, microtubule-associated protein 2 (MAP2), neurogranin [5] and interestingly BDNF itself [19].

In this study we explore this convergence of three inputs: BDNF, MAPK, and Ca^2+^ onto dendritic protein synthesis. We first parameterize the complex BDNF signaling pathway in stages, using published experimental data. We fold in previously published models of Ca^2+^ and MAPK signaling, and check that the combined model is consistent with further experiments. With the combined model we explore a range of input combinations and analyze how the positive feedback in the system may influence its behavior.

## Results

Our study was in two parts: parameterization and prediction. We first parameterized the BDNF signaling cascade using three sub-modules for BDNF receptor signaling, AKT, and S6 Kinase each with their own experimental inputs (Figure 1). We then modeled protein synthesis as a further set of three modules: 4E-BP (eIF4E release), initiation regulation by eIF4E (formation of 40S complex), and eEF2 regulation by CaMKIII. Then we merged in three existing models for synaptic signaling (Calmodulin (CaM), Protein kinase C (PKC) and MAPK) and tested the overall model. Parameterization was primarily based on published experiments reporting direct kinetic constants, or derivations of such constants from time-course experiments and activation ratios. These steps are presented in detail in the supplementary material (Dataset S1). The model was constrained at various steps by reproducing more complex experiments where measurements were made of intermediate molecular species *in vivo* or in cell extracts. In each of these cases, the underlying model was unchanged except for the concentrations of input molecules, whose values were set according to the protocol of the experiment that we were reproducing. This was an iterative process designed to obtain a reasonable fit of the model to a wide range of experiments involving sub-parts of the system as well as the whole. In the parameterization sections, we show the pathway chemical diagram with dashed ovals to represent input and output molecules, and shaded ovals to indicate molecules monitored in the constraining simulations.

The prediction part of the study used this composite model to analyze the dependence of protein synthesis on combinations of inputs relevant to synaptic plasticity. We examined steady-state responses and responses to typical LTP and LTD-inducing stimuli. We finally analyzed feedback effects in the system to predict the conditions under which the pathway might exhibit bistability.

### Module 1: BDNF Receptor Signaling

#### Pathway summary

**Figure 2:**
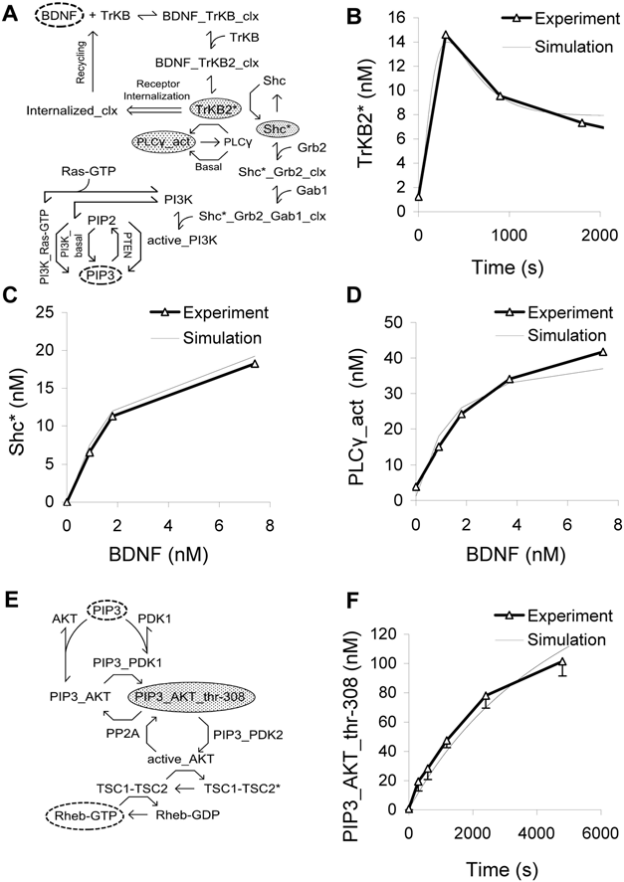
BDNF and AKT signaling. (A) Chemical reaction diagram of BDNF input module, terminating on PIP3 production. Molecules in shaded gray ovals are used as readouts for constraining the module. (B) Time-course of TrKB2 phosphorylation following BDNF (200 ng/ml) stimulation [28]. (C) Dose-response of Shc phosphorylation as a function of BDNF stimulus [28]. (D) Dose-response of PLC-γ phosphorylation as a function of BDNF stimulus [28]. (E) Chemical reaction diagram of AKT module, terminating in Rheb-GTP formation. (F) Time-course of AKT phosphorylation following PIP3 input [35].

BDNF activates TrkB, leading to stimulation of PI3K and formation of PIP3 (Figure 2A).

#### Background

BDNF is a ∼274 AA long dimeric neurotrophin that binds to tropomyosin-related kinase B (TrkB), a tyrosine kinase B receptor and thus induces dimerization and autophosphorylation of receptor. The phosphorylated receptor is involved in PI3K and Phospholipase C-γ (PLC-γ) activation (Figure 2A) [20],[21].

Phosphorylated TrKB receptor directly phosphorylates PLC-γ, thus activates it [20]. The mechanism of PI3K activation is somewhat elusive. *In vitro* studies show activation of PI3K by direct association between phosphorylated cytoplasmic tail of TrkB and the src homology 2 (SH2) domain of noncatalytic p85 subunit of PI3K [21] but *in vivo* this is not observed [22],[23]. Therefore we did not model this association. Instead, we based our model on *in vivo* data showing that phosphorylated TrKB receptor induces Shc adaptor protein phosphorylation [20],[24]. We modeled PI3K activation via three activator proteins: Shc, Grb-2 and Gab-1, as follows. Phosphorylated Shc binds to Grb-2 and then forms a complex by binding to a proline-rich region in the docking protein Gab1 [25],[26]. This complex binds to PI3K and activates it. PI3K is also activated by binding to Ras-GTP [27]. Active PI3K phosphorylates PtdIns(3,4)*P*2 (PIP2) to PtdIns(3,4,5)*P*3 (PIP3) which is then dephosphorylated by PTEN (phosphatase and tensin homologue).

#### Model constraints

Following parameterization using published rate constants (Dataset S1), the model was tuned against three published experiments. In the first experiment, NIH3T3 cells were stimulated with BDNF (200 ng/ml) for different periods. Phosphorylated TrKB was measured using western blots [28]. To simulate these experiments we ran the basal model to steady-state, and then added 200 ng/ml BDNF. We monitored the time course of the phosphorylated TrKB species in the model (Figure 2B). We also fitted our model to a concentration-effect curve for Shc and PLC-γ phosphorylation downstream of BDNF signal [28]. In the experiment, BDNF was applied to NIH3T3 cells for 5 minutes. At the end of 5 minutes, Shc and PLC-γ phosphorylation were measured using Western blots. The same input was used for the simulation, and the amount of phosphorylated Shc and PLC-γ was recorded (Figure 2C and 2D).

### Module 2: AKT Signaling

#### Pathway summary

PIP3 activates AKT (also known as Protein Kinase B, (PKB)) leading to accumulation of Rheb-GTP (Ras homolog enriched in brain-guanosine triphosphate), a regulator of TOR (Figure 2E).

#### Background

AKT needs to be doubly phosphorylated to be active. Both stages occur at the membrane. PIP3 binds to AKT and recruits it to the membrane, and does the same for PDK1. In the membrane, the PDK1-PIP3 complex phosphorylates AKT on thr308 [29]. There is a further sequential phosphorylation step where AKT is phosphorylated on ser-473 by PIP3-PDK2 [30]. Both these phosphoryations are reversed by protein phosphatase 2A (PP2A). The doubly phosphorylated AKT is active and phosphorylates TSC1-TSC2 (Tuberous sclerosis 1-Tuberous sclerosis 2) which in turn regulates Rheb-GTP levels. In its basal state TSC2 is present in a heterodimeric complex with TSC1 (also known as hamartin) known as hamartin-tuberin complex (TSC1-TSC2) [31]. The unphosphorylated complex has GTPase-activating protein (GAP) properties towards Rheb, a small GTPase [32]. TSC1-TSC2 hydrolyzes Rheb-GTP to Rheb-GDP. The net effect of these steps is that the active AKT inactivates TSC1-TSC2, and thus Rheb-GTP remains high. Overall, PIP3 activates AKT leading to a net activation of the downstream target, TOR.

The above model assumes that PDK1 activity does not change. We also considered the observation that high frequency stimulation in hippocampal neurons leads to the activation of PDK1. It is known that MAPK activates PDK1 [33]. We modeled the activation of PDK1 by active MAPK and binding of active PDK1 to PIP3 in an alternate model (Figure S1A and S1B). We delivered a steady 3.7 nM BDNF stimulus at 6000 sec, and compared the activation of AKT of this model with the reference model (model without PDK1 activation) (Figure S1C). The AKT response in the two models was similar, although there were quantitative differences in some other downstream molecules (Figure S1D–F). This result replicates experimental observations from previous studies which suggest that the activation of PDK1 by MAPK does not have a large effect on AKT phosphorylation [34]. We did not further consider the alternate model with MAPK activation of PDK1.

#### Model constraints

We modeled a published time-series experiment where AKT and PDK1 were incubated in presence of ATP and PIP3 for varying times, and the amount of phosphate incorporated in AKT was assayed using specific antibodies [35] (Figure 2F). To model this experiment, we initialized PIP3, AKT, and PDK1 at experimental levels (0.25 µM, 0.5 µM, 12 U/ml (0.0035 µM) respectively). We then ran the simulation and recorded the amount of phosphorylated AKT as a function of time (Figure 2F).

### Module 3: S6 Kinase Signaling

#### Pathway summary

**Figure 3:**
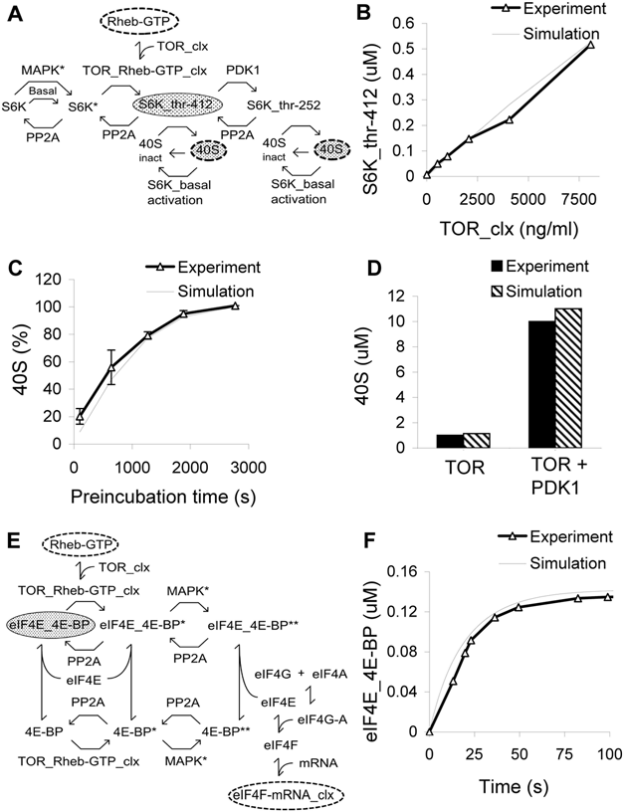
TOR signaling. (A) Chemical reaction diagram of S6K regulation culminating in 40S phosphorylation. (B) S6K phosphorylation as a function of TOR levels [44]. (C) 40S phosphorylation as a function of preincubation time of TOR and S6K (0.5, 10, 20, 30, and 45 min) [37]. (D) 40S phosphorylation is stimulated 10 fold by PDK1 [37]. (E) Chemical reaction diagram of eIF4E regulation, culminating in formation of the eIF4F-mRNA complex. (F) Time-course of eIF4E-BP complex formation [47].

Rheb-GTP activates TOR, and along with convergent input from MAPK, leads to S6K activation and formation of the active 40S for translation (Figure 3A).

#### Background

Rheb-GTP binds to TOR_clx (Target of rapamycin complex) to stimulate its kinase activity [36]. S6 Kinase (S6K) undergoes sequential phosphorylation by several kinases including MAPK* (at Ser-434, Ser-441, Ser-447, Ser-452, and Thr-444), TOR complex (at Thr-412 and Thr-444/Ser-447) and PDK1 (at Thr-252) [37] (Figure 3A). We modeled this phosphorylation cascade. The first phosphorylation step is regulated by several kinases in addition to MAPK (e.g., SAP kinases, p38s and Cdc2) [38]. These additional kinases are activated by their own signaling cascades, and are out of the scope of our study. For this study we explicitly modeled only the MAPK activation cascade, and represented the other kinases as a single kinase activity controlled by external regulatory inputs.

The second phosphorylation step is mediated by TOR. TOR is a complex of mTOR, LST8 and Raptor [39],[40]. The kinase activity of the TOR complex increases when it is bound to Rheb-GTP [36]. We modeled S6K phosphorylation by this bound complex.

The third phosphorylation step is mediated by PDK1, which we modeled as a constant kinase activity. As discussed above, we also modeled an alternate PDK1 model where MAPK stimulated PDK1 activity. For completeness, we also compared S6 Kinase and downstream activities of this alternate model with the reference model, and again there was little difference (Figure S1D–F).

We included dephosphorylation of all of these sites by PP2A [41]. The double and triple-phosphorylated forms of S6K are active and phosphorylate S6, which is a subunit of the 40S ribosomal protein. We modeled phosphorylation of the 40S by both the double and triple-phosphorylated S6K, as well as by a basal level of S6K activity.

There are reports that eukaryotic initiation factor 4B (eIF4B) contributes to protein synthesis in the absence of active S6 [42]. A possible alternative input is via ribosomal S6 kinase (RSK), which also phosphorylates eIF4B [43]. Due to lack of sufficient biochemical data about the regulation of RSK by MAPK, we did not model this reaction.

#### Model constraints

We carried out three tests of this module. First, we compared results for phosphorylation of S6K on thr-412, as a function of TOR levels [44] (Figure 3B). In these experiments, 500–8000 ng/mL of TOR was added to S6K (1.2 µM) and incubated for 1 hr. S6K phosphorylation was assayed using specific antibodies. The simulation duplicated this manipulation, and measured the level of phosphorylated S6K. Second, we modeled a time-course experiment where TOR and S6K were incubated for 0.5, 10, 20, 30, and 45 min. PDK1 was set to zero. 40S was then added for 15 min before reaction termination. The samples were analyzed by autoradiography. Phosphorylated 40S was quantified by image analyzer and plotted against incubation time [37]. The simulation matches the time-course of phosphorylation of 40S to within the error bars, with the exception of the first data point (Figure 3C). The third experiment reported a further 10-fold increase of S6K activity measured by autoradiography over the TOR-phosphorylated state, upon addition of PDK1 to the TOR-S6K reaction mixture. TOR and S6K were incubated for 0.5, 10, 20, 30, and 45 min. To this mixture PDK1 was added for 30 min, and then 40S was added for 15 min [37]. The level of phosphorylated 40S was quantified by image analyzer. The simulation replicated this experiment and produced similar levels of phosphorylated 40S (Figure 3D).

### Module 4: 4E-BP Signaling

#### Pathway summary

Rheb-GTP activates TOR, again with regulation by MAPK, leading to formation of the eIF4F-mRNA complex (Figure 3E).

#### Background

In the basal state, 4E-BP forms a complex with eIF4E. This prevents eIF4E from binding to eukaryotic initiation factor 4A (eIF4A) and eukaryotic initiation factor 4G (eIF4G) and thus inhibits the formation of eukaryotic initiation factor 4F (eIF4F). There are various views on the release of 4E-BP. One viewpoint is that phosphorylation of 4E-BP Ser65 site leads to dissociation of eIF4E_4E-BP [45]. An alternative model is that the formation of the complex protects the ser65 site from phosphorylation by MAPK. Our model is based on the latter view, where eIF4E_4E-BP is phosphorylated by the active TOR complex at thr-37 and thr-46 site. These phosphorylation steps are followed by dissociation of eIF4E_4E-BP and phosphorylation by MAPK* [46]. eIF4A and eIF4G binds and form a eIF4G-eIF4A complex which then binds to released eIF4E to form eIF4F. eIF4F in turn binds to mRNA to form eIF4F-mRNA complex.

#### Model constraints

We reproduced an experiment that monitored binding of eIF4E to 4EBP, using surface plasma resonance. 4EBP was immobilized on nickel-coated chips [47] (Figure 3F). The simulation simply modeled the time-course of association of eIF4E and 4EBP.

### Module 5: CaMKIII Signaling

#### Pathway summary

**Figure 4:**
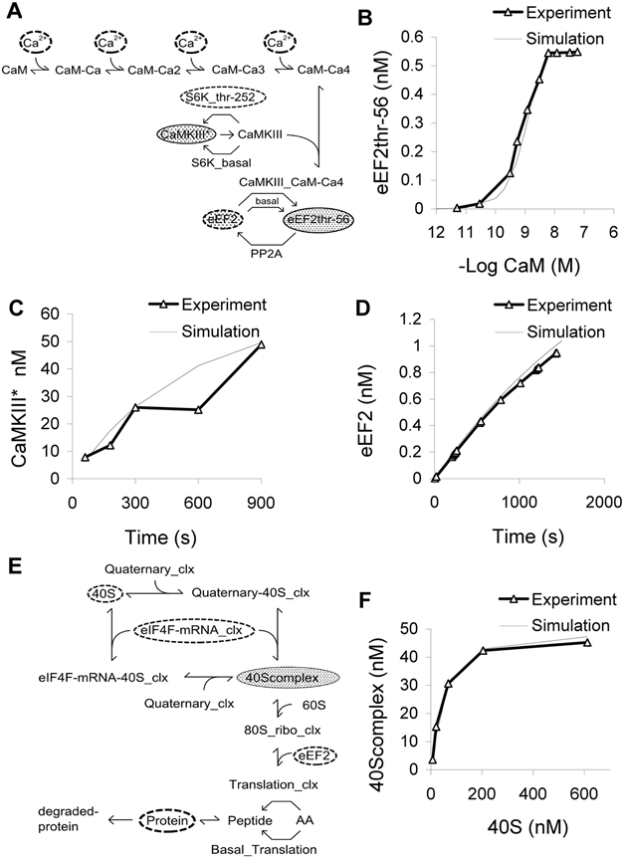
CaMKIII and translation complex modules. (A) Chemical reaction diagram of CaMKIII activation by Ca^2+^, culminating in eEF2 inhibition. (B) Dose-response curve for activation of CaMKIII by CaM-Ca4, as measured by eEF2 phosphorylation [51]. (C) Time-course of CaMKIII phosphorylation following S6 Kinase incubation [50]. (D) Time-course of eEF2-thr36 dephosphorylation following PP2A incubation [49]. (E) Chemical reaction diagram of final stages of translation complex formation, leading to protein synthesis. (F) Dose-response curve for 40S complex formation as a function of 40S concentration [54].

CaMKIII is activated by Ca^2+^ and inactivated by S6K. Since CaMKIII inhibits eEF2, the net effect of Ca^2+^ on elongation is inhibitory and of S6K is excitatory (Figure 4A).

#### Background

eEF2 is the major substrate for CaMKIII (also known as eEF2K, eEF2 kinase) [48]. CaMKIII catalyzes eEF2 phosphorylation at thr-56 and thr-58, strictly in the presence of Ca^2+^ and CaM [49]. CaMKIII phosphorylates eEF2 to render it inactive. This is one of the major Ca^2+^ inputs to our model.

Dephosphorylation of eEF2 by PP2A restores its activity [49]. S6K phosphorylates CaMKIII at Ser-366 [50] and decreases its activity and thus helps in increasing the level of eEF2. Our model includes phosphorylation of eEF2 only at its thr-56 site because the literature shows that the amount of the bi-phosphorylated form is negligible (2%) compared to the mono-phosphorylated (59%) and unphosphorylated (39%) forms in normal reticulocyte cell lysate [49].

#### Model constraints

We simulated three experiments to check the CaMKIII pathway model. In the first experiment, the calmodulin dependence of CaMKIII activity was measured in terms of eEF2 phosphorylation [51] (Figure 4B). Different levels of CaM were added to a reaction mixture consisting of CaMKIII, Ca^2+^ and eEF2, and the mixture incubated for 5 min. To simulate this curve we used CaM-Ca4 as the input to the model and measured resulting levels of phosphorylated eEF2. In the second experiment, active S6K was added to CaMKIII and the time-course of CaMKIII phosphorylation was measured using SDS-PAGE [50] (Figure 4C). We repeated these manipulations in the simulation, and monitored the CaMKIII* (phosphorylated CaMKIII) species. There was a discrepancy with experiment at the 10-min time point, but we considered the smooth simulated increase to be plausible. In the third experiment, active eEF2-Thr-36 was incubated with PP2A and the release of radiolabeled ^32^P phosphate was measured with respect to time [49] (Figure 4D). In the corresponding simulation, we modeled the dephosphorylation reaction and directly measured the formation of unphosphorylated eEF2. The curves matched within 10%.

### Module 6: Protein Synthesis

#### Pathway summary

40S, the eIF4F-mRNA complex, and eEF2 bind to form the translation complex, leading to protein synthesis (Figure 4E).

#### Background

**Figure 5:**
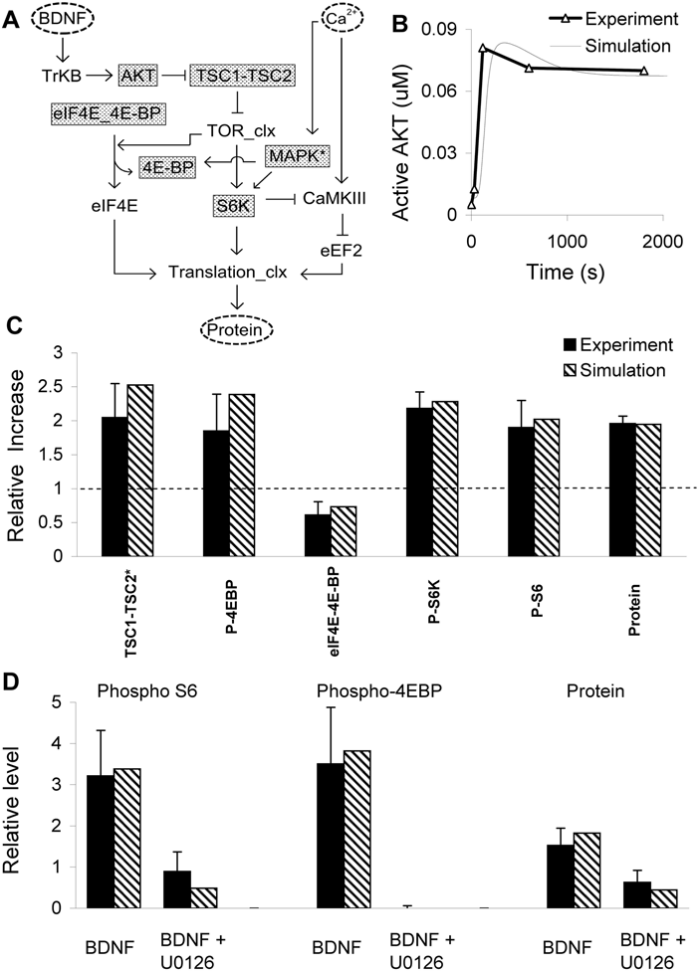
Composite pathway validation. (A) Simplified block diagram of composite pathway, showing key readout molecules. (B) Time-course of AKT activation following sustained BDNF stimulation at 2 nM [20]. (C) Relative increase in levels of various pathway readouts: TSC1-TSC2* (phosphorylated form of TSC1-TSC2), P-4EBP (sum total of the phosphorylated forms of 4E-BP), eIF4E-4E-BP (inactive form of eIF4E), P-S6K (sum total of the phosphorylated forms of S6K), P-S6 (phosphorylated form of 40S) and protein. The readouts except protein were measured after 10 minutes and protein was measured after 30 minutes following the addition of 3.7 nM BDNF [56]. The dotted line represents the value of read-outs without any stimulus (control) (D) Relative increase in levels of phosphoS6 (phosphorylated form of 40S), phospho-4EBP (doubly phosphorylated form of eIF4E_4E-BP), and protein, measured after 4 hr following 100 ng/mL BDNF stimulation for 4 hr, in control and MAPK KO mice [57].

Translation initiation is a key regulatory step in protein synthesis. We modeled the formation of the 40S initiation complex based on a published reaction scheme [52] which involves binding of 40S ribosomal subunit, mRNA and quaternary complex (a combination of eIF-2, GTP Met-tRNA and the factor Co-eIF-2C). We then modeled the binding of 40S initiation complex with 60S complex to form an 80S complex which in turn binds with eEF2 to form the translation complex. In our model this translation complex is represented as an enzyme that elongates and synthesizes protein. We selected parameters for this enzyme so that each ‘molecule’ of translation complex would produce a protein molecule in approximately 75 seconds. We have also included the basal synthesis of protein to account for synthesis of proteins independent of mTOR [53]. In all the calculations in this study, except for those discussed specifically in the Feedback and Bistability section, the synthesis and degradation processes acted only on a separate molecular species. This species is labeled as ‘Protein’ in Figures 1, 4E, 5A, and 9A. We assumed that all other molecules were present at steady-state levels, i.e., that their synthesis and degradation were balanced through reactions outside the scope of our model. In the final section on Feedback and Bistability we couple synthesis and turnover to three specific molecules in the model.

We lacked direct data for the degradation rates for most proteins. We therefore assigned a generic degradation rate of 1/sec for our synthesized ‘Protein’ species, for convenience. Thus,
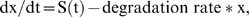
(1)where x is the concentration of the synthesized ‘Protein’ species and S(t) is the synthesis rate at time t.

If S(t) changes very slowly compared to the degradation rate, which is set to 1/sec, then the system settles to:
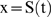
(2)Hence, the concentration x of synthesized ‘Protein’ is a readout of the synthesis rate.

With this rate, the concentration and synthesis rate of proteins are identical (barring units), provided that the synthesis rate changes on a much slower time-scale.

#### Model constraints

We reproduced an experiment that measured formation of 40S initiation complex as a function of 40S concentration. In the cellular translation process, the eIF4F-mRNA complex combines with the quaternary complex and 40S to form the 40S initiation complex. In the experiment, varying amounts of 40S were added to a mixture of quaternary complex and mRNA. Complex formation was measured by radioactivity of the filter-trapped complex. We assumed that the kinetics were independent of the association of eIF4F with mRNA. The levels of 40S initiation complex were within 10% of the experimental data [54] (Figure 4F).

### Pre-existing Modules: CaM and MAPK Signaling

We incorporated previously published models for CaM, PKC and MAPK (ERKII) signaling inputs to the current model [55] (Figure S2). These models have all been parameterized and matched with experimental data in our earlier work. The main input to these pathways in our model is Ca^2+^, which stimulates PKC directly. MAPK is downstream of PKC as well as CaM-Ca4 in our model, so MAPK activity is closely tied to synaptic activity leading to Ca^2+^ influx. Many additional inputs to MAPK are known but were not included in our model.

### Validation of the Composite Model

We merged all the sub-models mentioned above and tested our overall model. In contrast to the individual constraint steps for each sub-model, the composite model validation monitored signal flow through the entire cascade. We illustrate the pathway with a block diagram (Figure 5A), where the readout points are in gray. We first modeled a time-course experiment in primary cultures of rat cortical neurons, in which a sustained 2 nM BDNF stimulus was applied in the bath, leading to activation of AKT (Figure 5B) [20]. The simulated time-course was slightly slower than the experimental time-course. We attributed this to the difference in temperatures between the constraining experiments used for our model (∼30 degrees C) and the temperature of this experiment (∼37 degrees C). We then modeled the published experiments which measured the relative increase in several downstream readouts and protein concentration following steady BDNF stimulation at 3.7 nM (100 ng/ml) [56]. Again, these experiments were carried out using primary cultures of rat cortical neurons at 37 degrees C. Therefore, we ran the simulations for twice as long (TSC1-TSC2*, P-4EBP, eIF4E-4E-BP, P-S6K, P-S6 for 10 minutes and protein for 30 minutes) as in the experiment (i.e. for 5 minutes and 15 minutes respectively) before measuring the readouts (Figure 5C). Finally, we validated our model against experiments carried out in primary hippocampal cultures from control and MAPK knockout mice, again at 37 degrees C [57]. These experiments measured intermediates and protein synthesis responses to BDNF stimulation. Our model was able to closely match the experimental observations (Figure 5D).

Overall, our composite model incorporated several individually constrained pathways, and we showed that the combined model was in agreement with more complex experiments that exercised a significant part of the protein synthesis regulatory cascade.

### Parameter Sensitivity Analysis

Our data sources were diverse, and despite our modular approach to parameter fitting, we had to estimate many rates indirectly. We therefore tested how parameter uncertainty affected model behavior by systematically varying each parameter and comparing responses with those of the original model. This approach reflects the robustness of the *in vivo* system where a small change in conditions does not lead to much change in its behavior, unless the change impinges on a key cellular control molecule.

**Figure 6:**
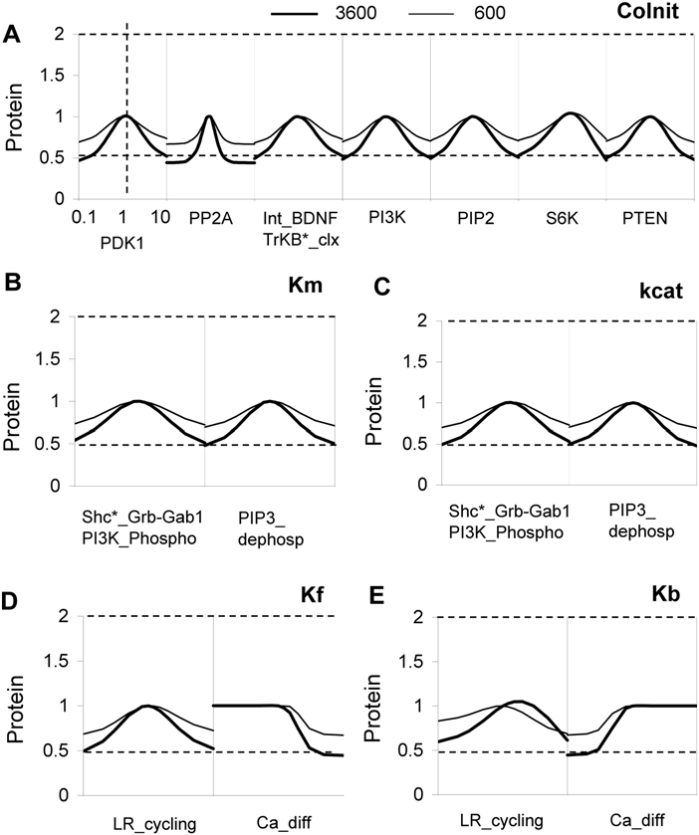
Parameter sensitivity analysis. We systematically varied all parameters from 0.1 to 10 fold the original model value. In all panels, the protein synthesis rate is plotted as a ratio to baseline, as measured at 600 sec (thin line) and 3600 sec (thick line). Here we plot the parameters that result in a change of at least a factor of two. (A) Dependence on initial concentration. (B) Dependence on Km, for enzyme reactions. (C) Dependence on kcat, for enzyme reactions. (D) Dependence on Kf, for binding or conversion reactions. (E) Dependence on Kb, for binding or conversion reactions. In all cases the synthesis rate does not change by more than 2.5 fold.

We systematically varied two enzyme parameters (Michaelis constant (Km) (Figure 6B) and turnover number (kcat)) (Figure 6C), two reaction parameters (forward rate (Kf) (Figure 6D) and backward rate (Kb) (Figure 6E)), and the total concentrations of each molecule (CoInit) (Figure 6A). Each parameter was varied over 2 log units, from 0.1 to 10 times the reference model value. As a readout we monitored the steady-state levels of PIP3_AKT-t308_s473 (active AKT), 4E-BP_tot (sum total of the phosphorylated forms of 4E-BP), MAPK*, S6K_tot (sum total of the phosphorylated forms of S6K) (Figures S3, S4, S5, S6, S7) and protein synthesis (Figure 6). Most parameters introduced only small changes in these readouts. A handful of parameters resulted in a two-fold or greater effect on responses, and these fell into two sets, corresponding to the BDNF and MAPK pathways of the model.

For the BDNF pathway, as measured by AKT activation, the major control molecules were PI3K, PTEN, PIP2 and PP2A. Most enzyme and binding reactions that were sensitive were also associated with these molecules. For the MAPK pathway, the control molecules were GEF, Ras, GAP, craf-1, PP2A and MKP-1. In addition to the reactions involving these molecules, the Ca^2+^ entry and PKC activation steps were sensitive control parameters.

**Figure 7:**
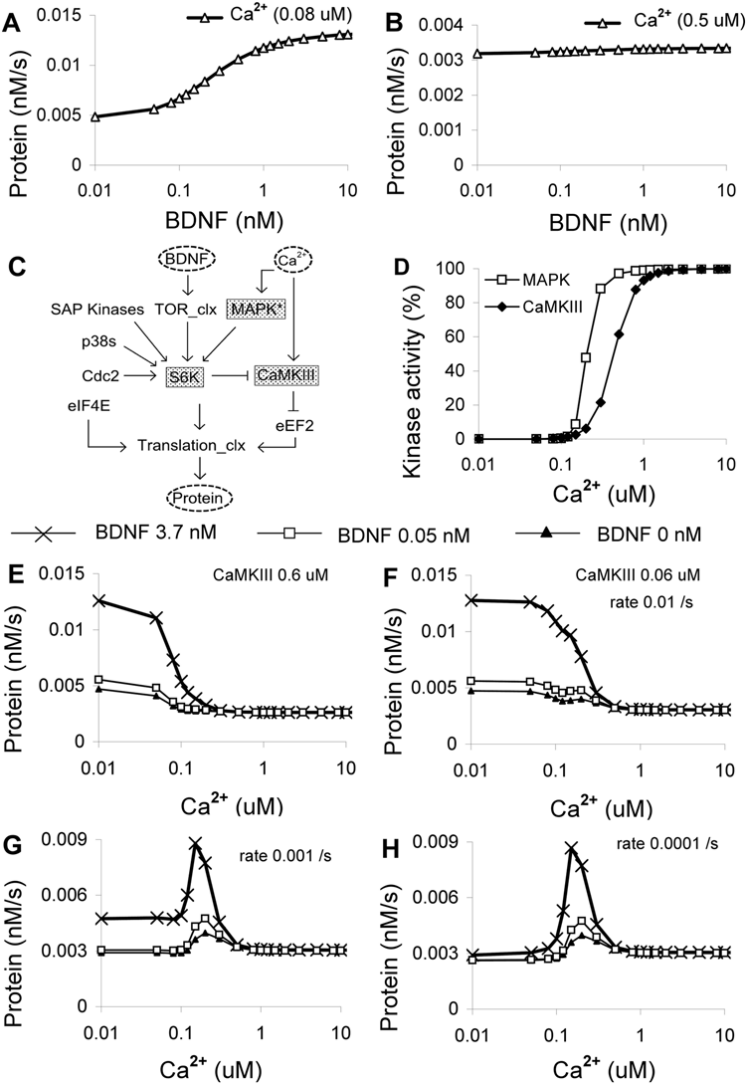
Steady-state responses to BDNF and Ca^2+^. (A,B) Protein synthesis rate as a function of BDNF level, at 0.08 and 0.5 µM Ca^2+^ respectively. (C) Simplified block diagram, indicating manipulated molecules in gray. (D) Comparison of activation curves for MAPK (phosphorylated form) and CaMKIII (CaM-Ca4 bound form) response to Ca^2+^. (E,F) Protein synthesis rates as a function of Ca^2+^, at two different levels of CaMKIII (0.6 µM (E), 0.06 µM (F)), both with a S6K phosphorylation rate of 0.01. There is a modest shift in Ca^2+^ dependence for the 3.7 nM BDNF case. (G,H) Reduced basal phosphorylation of S6K from 0.01 to 0.001 (G) or 0.0001 (H) converts the Ca^2+^ responses to a narrow bell-curve gated by BDNF.

A final key control parameter was the background rate of phosphorylation of S6K (S6K*) (Figure 7C). This is a convergence point for many kinases, including MAPK, SAP kinases, p38s and Cdc2 [38]. Of these, only MAPK is included in the present study. As discussed below, this phosphorylation step may act to switch the protein synthesis pathway into two distinct modes of responses to inputs.

### Steady-State Responses

In a first set of predictions of the composite model, we examined steady-state responses to BDNF and Ca^2+^ stimulation. The response to BDNF was a typical sigmoid but with a rather high baseline, so that the total increase in protein synthesis rate was just over two-fold (Figure 7A). High Ca^2+^ lowered the response and nearly flattened the sigmoid (Figure 7B). We then investigated why Ca^2+^ acted in this unexpected manner. From the reaction scheme, Ca^2+^ stimuli led to activation of two key kinases with opposing effects (Figure 7C). The MAPK cascade was activated through CaM-Ca4 as well as PKC. BDNF had no effect on MAPK responses. CaMKIII was also activated by CaM-Ca4, but at a higher Ca^2+^ concentration (Figure 7D). This opposing effect suggests that there may be a narrow window of Ca^2+^ in which synthesis is maximal. This narrow window gives rise to a bell-shaped curve when protein synthesis is plotted against Ca^2+^ levels. Interestingly, the peak of this bell curve is at rather low calcium levels, only about 2 or 3 fold basal levels. Mechanistically, this is because the differential region of the two Ca^2+^ activation curves (Figure 7C) is in this Ca^2+^ range. Physiologically, this Ca^2+^ range corresponds to mild rather than intense synaptic input [58]. In our default model, we found that high Ca^2+^ above 0.5 µM turned off protein synthesis (Figure 7E). As expected, higher levels of CaMKIII lowered this cutoff point (Figure 7F). Although these responses might suggest that strong Ca^2+^ influx (as in LTP stimulation) should block synthesis, the CaMKIII response is much faster than that of MAPK, and the system response to a Ca^2+^ transient may be more nuanced. We test this situation in the section on LTP and LTD stimuli.

One of the key regulatory input points to the protein synthesis cascade is S6K phosphorylation. S6K is known to be a target for other kinases, including SAP kinases, p38s and Cdc2 [38], in addition to MAPK and TOR (Figures 3A and 7C). In the model we had a steady basal kinase (BK) activity (rate is equal to 0.01 /sec) (Figure 7F) to represent the external kinase inputs. We now varied this BK activity to represent regulatory input. We found that the BK rate had a profound effect on the network controlling protein synthesis. At low BK, the network was in a state where protein synthesis was mostly low and gradually increased with an increase in MAPK activity. At high BK, the network was less susceptible to change in MAPK activity (Figure S8). Second, Ca^2+^ sensitivity switched from an inhibitory response above 0.5 µM Ca^2+^ to a BDNF-gated bell-shaped response (Figure 7G and 7H). Thus protein synthesis was high only when BDNF was high, and Ca^2+^ was in the 0.15 to 0.3 µM range. This unusual profile was due to high-sensitivity activation of MAPK by Ca^2+^, followed by the lower-sensitivity inhibition of protein synthesis via CaMKIII (Figure 7D).

Overall, we found that our composite model exhibited two possible kinds of behavior: BDNF gated and MAPK gated. The BDNF-gated behavior seemed more consistent with experiments in neuronal systems, and was contingent upon background phosphorylation of S6K by kinase regulation outside the scope of our study. In this mode BDNF had a strong effect on protein synthesis, and MAPK (stimulated by Ca^2+^ in our model) acted to further elevate responses. The MAPK-gated behavior may be of interest in other cell types [59],[60] or specific neuro-regulatory contexts [61]. It had a lower basal synthesis rate, a strong dependence on MAPK, and a narrow window of activation by Ca^2+^.

### LTP and LTD Stimuli

We next tested the model responses to temporal input activity patterns used for inducing LTP and LTD. We combined patterned Ca^2+^ input with varying levels of simulated BDNF to explore the effects of possible combinations of these inputs at the synapse. Based on the pathways in our model, BDNF should activate synthesis through AKT and TOR, whereas Ca^2+^ should turn on MAPK to activate synthesis, but also turn on CaMKIII to depress synthesis.

**Figure 8:**
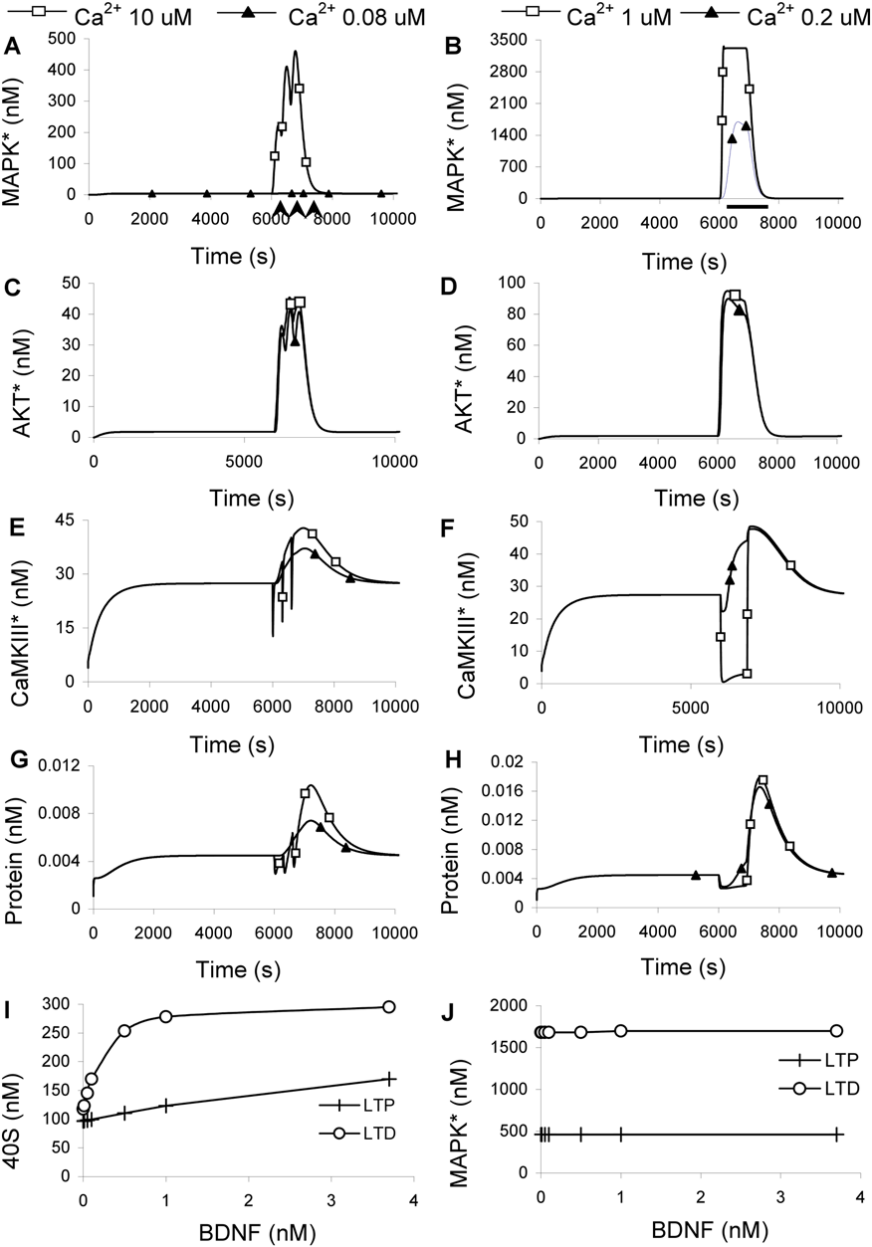
Responses to LTP and LTD stimuli. In (A–F) the LTP responses are on the left and LTD on the right. LTP stimulus was 3 Ca^2+^ peaks of 10 µM for 1 sec each, separated by 5 min, accompanied by BDNF input at 3.7 nM for 5 sec each (Arrows below time axis in (A)). Filled triangles indicate runs where the Ca^2+^ remained at baseline (0.08 µM) and only the BDNF stimulus was given. The LTD stimulus (Bar below time axis in (B)) was a single pulse of Ca^2+^ for 900 sec at 1 µM (open squares) and 0.2 µM Ca^2+^ (filled triangles). BDNF was a single 900 sec pulse at 3.7 nM. (A,B) MAPK activation. (C,D) AKT activation. (E,F) CaMKIII activation. (G,H) Protein synthesis. (I) Peak 40S as a function of BDNF levels for the LTD and LTP stimuli, at 0.2 and 10 µM Ca^2+^, respectively. The LTD stimulus gives nearly twice the 40S levels except at very low BDNF. (J) Peak MAPK activation by LTD and LTP stimuli, at 0.2 and 10 µM Ca^2+^, respectively. MAPK responds more strongly to the LTD stimulus, and is not sensitive to BDNF levels.

We represented the LTP stimulus as three pulses of Ca^2+^ influx, each 1 second wide, and separated by 300 sec [55]. In addition, we provided a BDNF input of 3.7 nM for 5 sec for each pulse of Ca^2+^ (Figure 8A). We monitored MAPK*, AKT* (PIP3_AKT_thr-308), CaMKIII*, and protein synthesis rate levels. The LTP stimulus caused a modest and brief elevation of protein synthesis rate through the combined action of MAPK and BDNF (Figure 8A, 8C, 8E, and 8G). We compared responses with three pulses of 10 µM Ca^2+^, and at basal Ca^2+^ (0.08 µM). We found that the MAPK response was entirely Ca^2+^ dependent. At basal Ca^2+^ CaMKIII and protein responses were indeed present, and were about half as large as for the 10 µM Ca^2+^ stimulus. This suggested that the contributions of the BDNF and Ca^2+^/MAPK inputs to protein synthesis were about equal.

We modeled LTD input as a single 900 sec Ca^2+^ pulse [62] along with a BDNF elevation to 3.7 nM for the same period. We again monitored MAPK*, AKT* (PIP3_AKT_thr-308), CaMKIII* and protein synthesis rate (Figure 8B, 8D, 8F, and 8H). Interestingly, the MAPK response was much stronger for LTD than for LTP stimulus, and the protein synthesis levels were nearly twice as large.

BDNF had exhibited gating behavior for steady-state responses, so we tested if it also did so for these transient stimuli. We ran additional simulations with zero BDNF, and found that though there were transients above and below baseline; neither the LTP nor LTD Ca^2+^ stimulus resulted in any net synthesis (Figure S9). Thus BDNF ‘gates’ LTP/LTD stimulated protein synthesis. While we did not model mGluR explicitly, it is known to converge onto AKT [18] which is in the BDNF pathway as well, and upstream of TOR in our model. Activation of TOR strongly enhanced the ability of both LTP and LTD stimuli to cause protein synthesis. Thus mGluR input might also act as a gate.

In contrast to the steady-state results, the large but brief Ca^2+^ transients in HFS stimuli caused an increase in protein synthesis provided BDNF was present. Our simulations showed that the transient stimulus did cause a brief CaMKIII-mediated reduction in synthesis, but this inhibition was relieved as soon as Ca^2+^ returned to baseline (Figure 8E and 8F). The same Ca^2+^ transient also acted through the slower MAPK-eIF2E pathway, leading to an elevation of synthesis after the inhibition had subsided. Thus the protein synthesis cascade acted like a transient detector for Ca^2+^ inputs. There are also CaMKII-mediated inputs to protein synthesis which may provide further timing interactions, but we were unable to parameterize these mechanisms on the basis of current data [58].

The duration of protein synthesis for LTP and LTD stimuli was short: of the order of 30 minutes. This is much less than the time-scale of protein synthesis dependent plasticity responses. We consider this interesting discrepancy in the discussion.

How might LTP and LTD stimuli differentially affect protein synthesis? One possibility is the differential phosphorylation of S6 leading to the activation of the 40S subunit, which has been implicated in 5′TOP mRNA translation [5],[63]. Another selective pathway is the eIF4E phosphorylation, which is elevated for CAP protein production [64],[65]. It is believed that MAPK phosphorylates MAPK-interacting kinase (MNK) which in turn phosphorylates eIF4E [5], but our model did not include MNK. There was no direct estimate for eIF4E phosphorylation so we took active MAPK (which phosphorylates eIF4E) as readout for CAP-protein production.

We monitored peak levels of 40S and active MAPK complex (as an estimate of CAP-protein production) for LTP and LTD simulations, while varying the BDNF stimulation over the range 0 to 3.7 nM in each case. This exploration was necessary because we did not have direct data for BDNF release levels during different synaptic stimulus protocols. We found that the MAPK response was independent of BDNF (Figure 8J), but the 40S levels were strongly BDNF dependent (Figure 8I). Except for very low levels of BDNF, the LTD stimulus elicited a nearly 2-fold larger 40S response than the LTP stimulus.

Overall, we find that TOR activation by BDNF is a prerequisite for both LTP- and LTD-triggered protein synthesis in our model. Different activity patterns, and different levels of co-regulatory inputs such as BDNF, may lead to a spectrum of differential activation of 5′TOP mRNA translation.

### Feedback and Bistability

**Figure 9:**
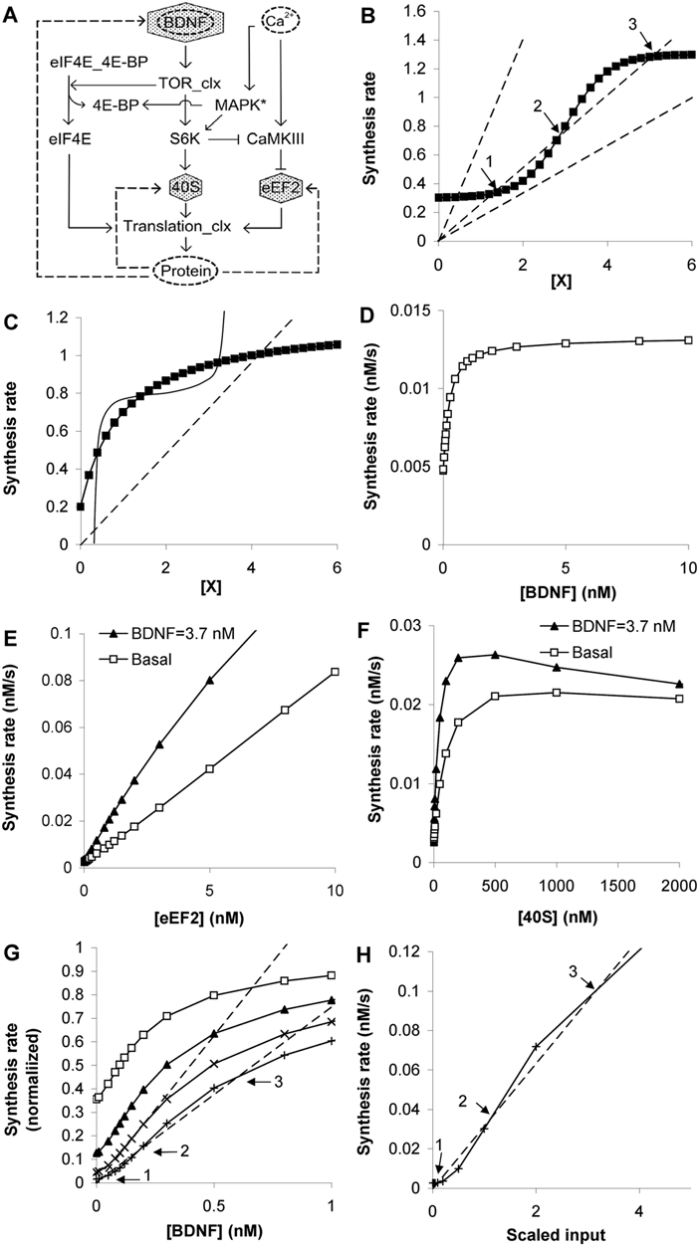
Feedback. (A) Three possible feedback pathways, indicated by dashed lines. 40S and eEF2 are produced by 5′TOP mRNAs and are involved in the protein synthesis machinery itself. BDNF is produced by the CAP mRNAs and is one of the key stimulus molecules for this pathway. (B) Bistability is possible with a steeply sigmoid dose-response curve (filled squares) if the input molecule X is a fraction of the total protein synthesis (dashed lines). This fraction F must be in an intermediate range such that the line cuts the sigmoid thrice (arrows). Points 1 and 3 are lower and upper stable points, and point 2 is an unstable point. (C) Bistability is not possible with a simple saturating dose-response curve (filled squares), when X is a fraction of protein synthesis (dashed line). It is only possible when X is itself formed in some steeply sigmoid manner as a function of synthesis rate, so as to give rise to three intersecting points (smooth line). (D) Protein synthesis rate as a function of BDNF stimulus. (E) Protein synthesis rate as a function of eEF2 levels, at basal (0.1 nM) and stimulated (3.7 nM) BDNF. (F) Protein synthesis rate as a function of 40S ribosomal subunit levels, again at basal and stimulated BDNF. None of (D), (E), or (F) can be bistable if the input molecule is produced in a linear proportion of total protein synthesis. (G) Bistability may be possible for 4^th^ order response curves. We normalized the BDNF response curve to a maximum of 1. We took powers of 1 to 4 of this curve (Squares, triangles, crosses and plus signs, respectively), and looked for sigmoidal shapes. The 3^rd^ power curve did not quite show bistability, but the 4^th^ power did (arrows as in (B)). (H) Synergistic effects of BDNF, eEF2 and 40S might show bistability (arrows as in (B)). We computed the dose response curve with scaled input (in nM) as follows: BDNF = input * 0.2; eEF2 = input * 1000, 40S = input * 100.

There are at least two forms of feedback possible in this system. First, protein synthesis may increase the production of the protein synthesis machinery itself. The ribosomal 40S subunit and the eEF2 protein are examples of such feedback molecules in our model (Figure 9A). Second, protein synthesis may increase the production of molecules, including BDNF, that contribute to activating synthesis (Figure 9A). BDNF is released from neurons in an activity-dependent manner, and recent studies show that some of this release may occur through postsynaptic mechanisms [66],[67]. Furthermore, there is evidence that BDNF has a postsynaptic site of action, in the induction of LTP [68]. Several studies have suggested that this release, coupled with the role of BDNF in synaptic plasticity, may constitute a feedback loop, and that at least part of this loop may be postsynaptic (reviewed in [69]). While it is likely that there is a combination of pre- and postsynaptic BDNF feedback in its overall action, for the purposes of our analysis we have considered only the postsynaptic component.

Both the protein synthesis machinery feedback, and the BDNF feedback have a positive sign, and are therefore suggestive of bistability [70],[71]. While 40 to 400 species of synaptic molecules have been reported to be synthesized at the dendrite [72], our model only included these three putative feedback molecules: BDNF, eEF2, and 40S. We first considered synthesis control of each of these individually, then in combination.

A simple graphical way to identify bistable systems arising from chemical feedback is to use overlaid dose-response curves [12],[73]. Intersection points on such curves indicate stable points of the system. If there are three intersection points (e.g., Figure 9B between the sigmoid and middle straight line) then the system is bistable. The lower intersection point is a stable state of low activity, and the upper intersection point a stable state of high activity. The intermediate intersection point has properties of a threshold or transition point between the two stable points. Note that these are steady-state curves, and the time taken to actually change state depends on reaction kinetics, the initial conditions, and on the kind of stimulus.

In our study the concentration of the synthesized protein (BDNF, eEF2 or 40S) is directly proportional to our readout of protein synthesis rate, assuming that molecular degradation is first order in concentration. In the following analysis, we therefore used the protein synthesis rate on the y axis as a surrogate for concentration. This has the advantage that two unknowns can be folded together into a single scaling factor: the degradation rate and the fraction of the specific protein out of the total protein synthesized. In the case of protein synthesis feedback, the simplest assumption was that a fixed fraction F of the synthesized protein could feedback to increase synthesis. Thus, protein concentration [X] may be represented as a straight line of slope 1/F through the origin (dashed lines in Figure 9B and 9C). It is clear from the geometries that a sigmoid dose-response curve may exhibit protein-synthesis bistability for some values of F (Figure 9B), but a logarithmic or saturating curve will never be bistable if it has a linear dependence on synthesis rate (dashed line in Figure 9C). The only situation in which a molecule with a saturating response curve might exhibit feedback bistability is if the level of protein is a steep sigmoid function of the synthesis rate (smooth line in Figure 9C).

We ran dose response curves for BDNF, the eEF2 protein and the ribosomal 40S subunit to look for bistability in this system (Figure 9D–F). None of the curves could support bistability with the linear feedback assumption (straight lines). In each case, there was just one intersection point between the dose-response curves, for any value of F. This was the case even if the system was co-stimulated with 3.7 nM BDNF (Figure 9E and 9F).

Having shown that individual synthetic pathways were unlikely to lead to bistability, we then asked if combinations might do so. We first considered higher-order activation processes, using the BDNF response as a typical dose-response curve. A hypothetical mechanism for this might be if the molecule acted in a higher-order manner to stimulate protein synthesis. We found that a fourth-order reaction of the form of the BDNF curve was sufficiently sigmoidal to just support bistability, but a third-order reaction was not (Figure 9G). As a more biologically grounded test, we considered if the known dendritically synthesized molecules BDNF, eEF2, and 40S might act synergistically to support bistability. We took an ‘optimum’ condition, where the protein synthesis scaling factor F for each was such that their half-maximal levels (K_half_) coincided. Using this scaling we re-ran the dose-response simulation, and found that the resultant curve was indeed sigmoidal and could support bistability for a narrow range of synthesis scaling factors (Figure 9H). As we discuss below, this bistability requires a finely tuned set of conditions to occur, but on the other hand may be strengthened by other, as yet unknown feedback reactions.

Overall, we conclude that a protein-synthesis switch at the dendrite is unlikely with currently known feedback mechanisms.

## Discussion

We have modeled the regulation of dendritic protein synthesis by three inputs: BDNF, MAPK, and activity leading to Ca^2+^ influx. Our model was closely constrained by many published experiments, and was in good agreement with known responses to a selection of stimuli. We found that protein synthesis was gated by BDNF and potentially mGluR acting through mTOR, and was differentially activated by LTP and LTD stimuli. Despite the positive feedback from the protein synthesis to synaptic signaling proteins, the system was unlikely to be bistable.

### Where Is the Switch?

Bistable switches are plausible mediators of long-term storage of information at a signaling level. Several such switches have been proposed to be involved in synaptic plasticity: CaMKII autophosphorylation [11], MAPK feedback loops [12],[13],[74], and alpha-amino-3-hydroxy-5-methyl-4-isoxazole propionate receptor (AMPAR) trafficking cycles [75], Our expectation for this pathway was that it might exhibit switch-like behavior of its own, resulting from positive feedback from synthesized proteins back into the synthesis machinery. However, we found that bistability could not occur if we used the simple assumption that the each feedback molecule was present at a fixed fraction of the total protein synthesized. We were able to achieve a fragile bistability when we assumed coordinated feedback effects, but only with considerable tuning of feedback processes. At face value this is extremely unlikely situation. Furthermore, an elevation of basal synthesis rates, or influx of some of the 5′TOP proteins from non-local synthesis, would shift the synthesis curves upward along the y-axis, and thus eliminate the bistability. As a counterpoint, however, it is possible that synergistic effects may occur due to further feedback effects of additional dendritically-synthesized proteins. Overall, we feel that protein-synthesis-feedback bistability is unlikely, but it will take considerable additional data to resolve this point.

While it seems reasonable that synaptic plasticity should draw upon new proteins and hence require elevated synthesis, recent studies suggest that the relationship between synthesis and plasticity is complex. Even though new synthesis is needed for plasticity, the total level of protein synthesis does not appear to change much following L-LTP stimuli [2]. Furthermore, background activity may actually act to suppress synthesis [76]. These observations further complicate the idea of protein-synthesis bistability in the dendrite.

### Model Scope and Unknowns

Our model of dendritic protein synthesis involves some 130 molecular species, including phosphorylation states and complexes of the key proteins. We have chosen this size model as a compromise between completeness and parameter unknowns. These concerns are present in all but the most exhaustively characterized signaling pathways [77],[78].

We have selected a subset of implicated pathways for this study based on their reported roles, and on the degree to which the pathways have been characterized. For example, we were not able to include the mGluR input to the pathway as it is still lacking key functional details [18]. Nevertheless, our integrated model validation results suggest that we have succeeded in capturing many of the key interactions for the processes we wished to study. The resulting model was rather insensitive to parameter variations. Even the ‘sensitive’ parameters, when scaled up or down tenfold, affected model responses by barely a factor of two. Key targets for future refinement of the model, besides mGluR input, would be a more complete analysis of differential protein synthesis, and incorporation of the kinases that phosphorylate S6K on its p70 carboxy-terminal tail [38].

A much more challenging goal is to model the turnover of every protein in the model, so that all of the molecular concentrations are the outcome of regulatory and homeostatic controls. This awaits substantial advances in our understanding of all stages of neuronal protein synthesis control.

### Self-Modifying Machines

The synapse is an unusual computational entity: it modifies its own hardware. Most computer languages manipulate data, but scrupulously avoid modifying program instructions. By these engineering standards, the synapse appears peculiar and unstable. In this system molecular signals (data) compute through chemical interactions (program instructions), which among other things also structurally modify the synapse and thereby change its computational properties. The protein synthesis cascade is an instance of this self-modification. How does the cell avoid runaway processes? Our study has some possible hints about how the system activity remains bounded. First, the synthetic increase is quite limited and the only way to get large fold activation over baseline is to provide synergistic BDNF and activity input. Thus, gating restricts the conditions where self-modification may occur. Second, our model suggests that the molecular logic of synthesis control may be somewhat insulated from the potentially dangerous process of positive feedback. Instead, potential feedback molecules seem to have saturating response curves with a high baseline that are effective at damping runaway buildup. With this interpretation, activity triggers leading to plasticity may initially activate biochemical switches rather than a protein synthesis loop. Candidate biochemical switches include CaMKII for fast switching, and MAPK or trafficking switches for slower phases of plasticity [12],[75],[79]. These switches, in turn, may regulate the protein synthesis pathway. Thus the short time-course of responses of the current model to synaptic input may simply reflect the role of synthesis as an effector of an upstream switch, rather than a switch itself. We speculate that very long-term changes may involve a shift in what is synthesized, and where it is made, rather than in how much synthesis occurs. To use our computational metaphor, this would be more like the machine loading different programs, rather than rebuilding and redesigning itself. This view would predict that very long-term plasticity involves decisions between a set of possible ‘programs’ executed by dendritic synthesis machinery to influence local synaptic function.

## Methods

Simulations were developed in GENESIS, the General Neuronal Simulation System, using the Kinetikit interface [80] and solved using the exponential Euler method. Later parameter sensitivity, dose-response, and time-course runs were done using MOOSE, the Multiscale Object-Oriented Simulation Environment (http://moose.sourceforge.net/) and solved using an adaptive Runge-Kutta method (GNU scientific library). Simulations were carried out on PC workstations and on a SUN/Opteron cluster running Linux. Complete model reaction schemes and parameters are presented in Dataset S2. To further check the model calculations, and to facilitate community access to our simulations, we converted our model to SBML Level 2 Version 1 (Dataset S3). We had to remove a tabulated BDNF-dependent calcium stimulus which is not supported by most simulators. We compared this slightly modified model with other simulators (e.g, COPASI 4.4 [81] and CellDesigner 4.0 [82] ) and obtained the same results as with GENESIS and MOOSE.

To validate our composite model, we ran the simulation for 6000 sec without any stimulus and then noted the concentration of TSC1-TSC2*, 4E-BP_tot, S6K_tot and total synthesized protein. We then applied a steady stimulus of 3.7 nM BDNF. In the experiments, the levels of TSC1-TSC2*, 4E-BP_tot and S6K_tot were noted at 5 min and the level of protein at 15 min. As our simulations used room-temperature rates, and these experiments were done at physiological temperatures, we doubled the runtimes for our simulations when carrying out these validations. The activated values were divided by the basal values to obtain fold activation.

Sensitivity analysis was done by scaling each parameter one at a time in the range of 0.1 to 10-fold of the original parameter values. The parameters were: initial concentration (for molecules with non-zero initial concentrations); Km and kcat (for enzymes); and Kf and Kb (for binding/conversion reactions). To measure sensitivity, we ran the scaled model for 6000s without any stimulus. At 6000s the value of BDNF was set at 3.7 nM and then the concentration of readouts was recorded at 6600 sec and 9600 sec. The concentration of the readouts (PIP3_AKT-t308_s473 (active AKT), 4E-BP_tot, MAPK*, S6K_tot and protein) were normalized by dividing the obtained concentration by the value obtained from the original parameter model. These normalized fold change were plotted against logarithmic value of the parameter scale factor to obtain the sensitivity plots.

## Supporting Information

Dataset S1 Parameters source and notes.
(0.72 MB XLS)

Dataset S2 Model equations and parameters.
(1.34 MB XLS)

Dataset S3 Model file.
(0.27 MB XML)

Figure S1 Comparison of responses of alternate model (model with MAPK activated PDK1 activity) with the reference model. Filled squares indicate responses from reference model and open triangles indicate responses from alternate model. Responses of : (A) PDK1, (B) PDK1*, (C) active AKT (PIP3_AKT_thr-308), (D) S6K_tot (sum total of the phosphorylated forms of S6K), (E) 40S (phosphorylated form of 40S), and (F) protein synthesis rate, after a delivery of steady stimulus of 3.7 nM BDNF.
(0.09 MB PDF)

Figure S2 Diagram of existed signaling models inputs to the current model (A) Chemical reaction diagram of published model of PKC regulation showing its activation (B) Chemical reaction diagram of published model of MAPK pathway.
(3.90 MB PDF)

Figure S3 Parameter sensitivity analysis. We systematically varied the total concentrations of each molecule (CoInit) from 0.1 to 10 fold the original model value. We plotted active AKT, MAPK*, S6K_tot (sum total of the phosphorylated forms of S6K) and 4E-BP_tot (sum total of the phosphorylated forms of 4E-BP).
(0.02 MB PDF)

Figure S4 Parameter sensitivity analysis. We systematically varied the Km of each molecule from 0.1 to 10 fold the original model value.
(0.01 MB PDF)

Figure S5 Parameter sensitivity analysis. We systematically varied the kcat of each molecule from 0.1 to 10 fold the original model value.
(0.01 MB PDF)

Figure S6 Parameter sensitivity analysis. We systematically varied the Kf of each molecule.
(0.01 MB PDF)

Figure S7 Parameter sensitivity analysis. We systematically varied the Kb of each molecule.
(0.01 MB PDF)

Figure S8 Dose response of protein synthesis rate as a function of active MAPK. BDNF is buffered at basal level (0.05 µM (A)) and at stimulated level (3.7 nM (B)).The basal kinase activity is 0.01 /sec. There is a weak dependence of protein synthesis rate on MAPK activity.
(0.08 MB PDF)

Figure S9 Response to LTP and LTD stimuli. (A) protein synthesis for the LTP stimuli and (B) protein synthesis for the LTD stimuli. LTP stimulus was 3 Ca^2+^ peaks of 10 µM for 1 sec each, separated by 5 min (Arrows below time axis in (A)). Filled triangles indicate runs where the Ca^2+^ remained at baseline (0.08 µM) and open squares indicate Ca^2+^ at 10 µM. The LTD stimulus (Bar below time axis in (B)) was a single pulse of Ca^2+^ for 900 sec at 1 µM (open squares) and 0.2 µM Ca^2+^ (filled triangles). The simulations are with zero BDNF.
(0.09 MB PDF)

## References

[1] Sutton MA, Schuman EM (2006). Dendritic protein synthesis, synaptic plasticity, and memory.. Cell.

[2] Schuman EM, Dynes JL, Steward O (2006). Synaptic regulation of translation of dendritic mRNAs.. J Neurosci.

[3] Tang SJ, Schuman EM (2002). Protein synthesis in the dendrite.. Philos Trans R Soc Lond B Biol Sci.

[4] Bramham CR, Wells DG (2007). Dendritic mRNA: transport, translation and function.. Nat Rev Neurosci.

[5] Klann E, Antion MD, Banko JL, Hou L (2004). Synaptic plasticity and translation initiation.. Learn Mem.

[6] Schuman EM, Seeburg PH (2006). Signalling mechanisms.. Curr Opin Neurobiol.

[7] Govindarajan A, Kelleher RJ, Tonegawa S (2006). A clustered plasticity model of long-term memory engrams.. Nat Rev Neurosci.

[8] Frey S, Frey JU (2008). ‘Synaptic tagging’ and ‘cross-tagging’ and related associative reinforcement processes of functional plasticity as the cellular basis for memory formation.. Prog Brain Res.

[9] Bhalla US (2003). Understanding complex signaling networks through models and metaphors.. Prog Biophys Mol Biol.

[10] Song H, Smolen P, Av-Ron E, Baxter DA, Byrne JH (2007). Dynamics of a minimal model of interlocked positive and negative feedback loops of transcriptional regulation by cAMP-response element binding proteins.. Biophys J.

[11] Lisman JE, McIntyre CC (2001). Synaptic plasticity: a molecular memory switch.. Curr Biol.

[12] Bhalla US, Iyengar R (1999). Emergent properties of networks of biological signaling pathways.. Science.

[13] Tanaka K, Augustine GJ (2008). A positive feedback signal transduction loop determines timing of cerebellar long-term depression.. Neuron.

[14] Xia Y, Carroll RC, Nawy S (2006). State-dependent AMPA receptor trafficking in the mammalian retina.. J Neurosci.

[15] Kang H, Schuman EM (1996). A requirement for local protein synthesis in neurotrophin-induced hippocampal synaptic plasticity.. Science.

[16] Huber KM, Kayser MS, Bear MF (2000). Role for rapid dendritic protein synthesis in hippocampal mGluR-dependent long-term depression.. Science.

[17] Wells DG, Dong X, Quinlan EM, Huang YS, Bear MF (2001). A role for the cytoplasmic polyadenylation element in NMDA receptor-regulated mRNA translation in neurons.. J Neurosci.

[18] Hou L, Klann E (2004). Activation of the phosphoinositide 3-kinase-Akt-mammalian target of rapamycin signaling pathway is required for metabotropic glutamate receptor-dependent long-term depression.. J Neurosci.

[19] Saarelainen T, Vaittinen S, Castren E (2001). trkB-receptor activation contributes to the kainate-induced increase in BDNF mRNA synthesis.. Cell Mol Neurobiol.

[20] Dijkhuizen PA, Ghosh A (2005). BDNF regulates primary dendrite formation in cortical neurons via the PI3-kinase and MAP kinase signaling pathways.. J Neurobiol.

[21] Obermeier A, Lammers R, Wiesmuller KH, Jung G, Schlessinger J (1993). Identification of Trk binding sites for SHC and phosphatidylinositol 3′-kinase and formation of a multimeric signaling complex.. J Biol Chem.

[22] Holgado-Madruga M, Moscatello DK, Emlet DR, Dieterich R, Wong AJ (1997). Grb2-associated binder-1 mediates phosphatidylinositol 3-kinase activation and the promotion of cell survival by nerve growth factor.. Proc Natl Acad Sci U S A.

[23] Ohmichi M, Decker SJ, Saltiel AR (1992). Activation of phosphatidylinositol-3 kinase by nerve growth factor involves indirect coupling of the trk proto-oncogene with src homology 2 domains.. Neuron.

[24] Huang EJ, Reichardt LF (2001). Neurotrophins: roles in neuronal development and function.. Annu Rev Neurosci.

[25] Schlessinger J, Lemmon MA (2003). SH2 and PTB domains in tyrosine kinase signaling.. Sci STKE.

[26] Patapoutian A, Reichardt LF (2001). Trk receptors: mediators of neurotrophin action.. Curr Opin Neurobiol.

[27] Kodaki T, Woscholski R, Hallberg B, Rodriguez-Viciana P, Downward J (1994). The activation of phosphatidylinositol 3-kinase by Ras.. Curr Biol.

[28] Yuen EC, Mobley WC (1999). Early BDNF, NT-3, and NT-4 signaling events.. Exp Neurol.

[29] Chan TO, Tsichlis PN (2001). PDK2: a complex tail in one Akt.. Sci STKE.

[30] Koh G, Teong HF, Clement MV, Hsu D, Thiagarajan PS (2006). A decompositional approach to parameter estimation in pathway modeling: a case study of the Akt and MAPK pathways and their crosstalk.. Bioinformatics.

[31] McManus EJ, Alessi DR (2002). TSC1-TSC2: a complex tale of PKB-mediated S6K regulation.. Nat Cell Biol.

[32] Inoki K, Li Y, Xu T, Guan KL (2003). Rheb GTPase is a direct target of TSC2 GAP activity and regulates mTOR signaling.. Genes Dev.

[33] Tsokas P, Ma T, Iyengar R, Landau EM, Blitzer RD (2007). Mitogen-activated protein kinase upregulates the dendritic translation machinery in long-term potentiation by controlling the mammalian target of rapamycin pathway.. J Neurosci.

[34] Zheng F, Soellner D, Nunez J, Wang H (2008). The basal level of intracellular calcium gates the activation of phosphoinositide 3-kinase-Akt signaling by brain-derived neurotrophic factor in cortical neurons.. J Neurochem.

[35] Alessi DR, James SR, Downes CP, Holmes AB, Gaffney PR (1997). Characterization of a 3-phosphoinositide-dependent protein kinase which phosphorylates and activates protein kinase Bα.. Curr Biol.

[36] Long X, Lin Y, Ortiz-Vega S, Yonezawa K, Avruch J (2005). Rheb binds and regulates the mTOR kinase.. Curr Biol.

[37] Isotani S, Hara K, Tokunaga C, Inoue H, Avruch J (1999). Immunopurified mammalian target of rapamycin phosphorylates and activates p70 S6 kinase α in vitro.. J Biol Chem.

[38] Alessi DR, Kozlowski MT, Weng QP, Morrice N, Avruch J (1998). 3-Phosphoinositide-dependent protein kinase 1 (PDK1) phosphorylates and activates the p70 S6 kinase in vivo and in vitro.. Curr Biol.

[39] Hara K, Maruki Y, Long X, Yoshino K, Oshiro N (2002). Raptor, a binding partner of target of rapamycin (TOR), mediates TOR action.. Cell.

[40] Kim DH, Sarbassov DD, Ali SM, Latek RR, Guntur KV (2003). GβL, a positive regulator of the rapamycin-sensitive pathway required for the nutrient-sensitive interaction between raptor and mTOR.. Mol Cell.

[41] Ballou LM, Jeno P, Thomas G (1988). Protein phosphatase 2A inactivates the mitogen-stimulated S6 kinase from Swiss mouse 3T3 cells.. J Biol Chem.

[42] Hay N, Sonenberg N (2004). Upstream and downstream of mTOR.. Genes Dev.

[43] Shahbazian D, Roux PP, Mieulet V, Cohen MS, Raught B (2006). The mTOR/PI3K and MAPK pathways converge on eIF4B to control its phosphorylation and activity.. EMBO J.

[44] Toral-Barza L, Zhang WG, Lamison C, Larocque J, Gibbons J (2005). Characterization of the cloned full-length and a truncated human target of rapamycin: activity, specificity, and enzyme inhibition as studied by a high capacity assay.. Biochem Biophys Res Commun.

[45] Karim MM, Hughes JM, Warwicker J, Scheper GC, Proud CG (2001). A quantitative molecular model for modulation of mammalian translation by the eIF4E-binding protein 1.. J Biol Chem.

[46] Gingras AC, Gygi SP, Raught B, Polakiewicz RD, Abraham RT (1999). Regulation of 4E-BP1 phosphorylation: a novel two-step mechanism.. Genes Dev.

[47] Ptushkina M, von der Haar T, Karim MM, Hughes JM, McCarthy JE (1999). Repressor binding to a dorsal regulatory site traps human eIF4E in a high cap-affinity state.. EMBO J.

[48] Ryazanov AG, Shestakova EA, Natapov PG (1988). Phosphorylation of elongation factor 2 by EF-2 kinase affects rate of translation.. Nature.

[49] Redpath NT, Price NT, Severinov KV, Proud CG (1993). Regulation of elongation factor-2 by multisite phosphorylation.. Eur J Biochem.

[50] Wang X, Li W, Williams M, Terada N, Alessi DR (2001). Regulation of elongation factor 2 kinase by p90(RSK1) and p70 S6 kinase.. EMBO J.

[51] Mitsui K, Brady M, Palfrey HC, Nairn AC (1993). Purification and characterization of calmodulin-dependent protein kinase III from rabbit reticulocytes and rat pancreas.. J Biol Chem.

[52] Manchester KL (1997). Binding constants in the formation of mammalian protein synthesis initiation complexes and the role of mRNA.. Biochem Biophys Res Commun.

[53] Wang X, Proud CG (2006). The mTOR pathway in the control of protein synthesis.. Physiology (Bethesda).

[54] Parkhurst KM, Hileman RE, Saha D, Gupta NK, Parkhurst LJ (1994). Thermodynamic characterization of the cooperativity of 40S complex formation during the initiation of eukaryotic protein synthesis.. Biochemistry.

[55] Ajay SM, Bhalla US (2004). A role for ERKII in synaptic pattern selectivity on the time-scale of minutes.. Eur J Neurosci.

[56] Takei N, Inamura N, Kawamura M, Namba H, Hara K (2004). Brain-derived neurotrophic factor induces mammalian target of rapamycin-dependent local activation of translation machinery and protein synthesis in neuronal dendrites.. J Neurosci.

[57] Kelleher RJ, Govindarajan A, Jung HY, Kang H, Tonegawa S (2004). Translational control by MAPK signaling in long-term synaptic plasticity and memory.. Cell.

[58] Atkins CM, Davare MA, Oh MC, Derkach V, Soderling TR (2005). Bidirectional regulation of cytoplasmic polyadenylation element-binding protein phosphorylation by Ca^2+^/calmodulin-dependent protein kinase II and protein phosphatase 1 during hippocampal long-term potentiation.. J Neurosci.

[59] Marshall CJ (1995). Specificity of receptor tyrosine kinase signaling: transient versus sustained extracellular signal-regulated kinase activation.. Cell.

[60] Treisman R (1996). Regulation of transcription by MAP kinase cascades.. Curr Opin Cell Biol.

[61] Waskiewicz AJ, Cooper JA (1995). Mitogen and stress response pathways: MAP kinase cascades and phosphatase regulation in mammals and yeast.. Curr Opin Cell Biol.

[62] Dudek SM, Bear MF (1992). Homosynaptic long-term depression in area CA1 of hippocampus and effects of N-methyl-d-aspartate receptor blockade.. Proc Natl Acad Sci U S A.

[63] Kleijn M, Scheper GC, Voorma HO, Thomas AA (1998). Regulation of translation initiation factors by signal transduction.. Eur J Biochem.

[64] Huang S, Houghton PJ (2001). Mechanisms of resistance to rapamycins.. Drug Resist Updat.

[65] Kanhema T, Dagestad G, Panja D, Tiron A, Messaoudi E (2006). Dual regulation of translation initiation and peptide chain elongation during BDNF-induced LTP in vivo: evidence for compartment-specific translation control.. J Neurochem.

[66] Kolarow R, Brigadski T, Lessmann V (2007). Postsynaptic secretion of BDNF and NT-3 from hippocampal neurons depends on calcium calmodulin kinase II signaling and proceeds via delayed fusion pore opening.. J Neurosci.

[67] Lu Y, Christian K, Lu B (2008). BDNF: a key regulator for protein synthesis-dependent LTP and long-term memory?. Neurobiol Learn Mem.

[68] Kovalchuk Y, Hanse E, Kafitz KW, Konnerth A (2002). Postsynaptic induction of BDNF-mediated long-term potentiation.. Science.

[69] Lessmann V, Gottmann K, Malcangio M (2003). Neurotrophin secretion: current facts and future prospects.. Prog Neurobiol.

[70] Blitzer RD, Iyengar R, Landau EM (2005). Postsynaptic signaling networks: cellular cogwheels underlying long-term plasticity.. Biol Psychiatry.

[71] Lisman JE, Fallon JR (1999). What maintains memories?. Science.

[72] Eberwine J, Miyashiro K, Kacharmina JE, Job C (2001). Local translation of classes of mRNAs that are targeted to neuronal dendrites.. Proc Natl Acad Sci U S A.

[73] Ferrell JE (2008). Feedback regulation of opposing enzymes generates robust, all-or-none bistable responses.. Curr Biol.

[74] Ferrell JE (2002). Self-perpetuating states in signal transduction: positive feedback, double-negative feedback and bistability.. Curr Opin Cell Biol.

[75] Hayer A, Bhalla US (2005). Molecular switches at the synapse emerge from receptor and kinase traffic.. PLoS Comput Biol.

[76] Marin P, Nastiuk KL, Daniel N, Girault JA, Czernik AJ (1997). Glutamate-dependent phosphorylation of elongation factor-2 and inhibition of protein synthesis in neurons.. J Neurosci.

[77] Bray D, Levin MD, Lipkow K (2007). The chemotactic behavior of computer-based surrogate bacteria.. Curr Biol.

[78] Shimizu TS, Bray D (2002). Modelling the bacterial chemotaxis receptor complex.. Novartis Found Symp.

[79] Mullasseril P, Dosemeci A, Lisman JE, Griffith LC (2007). A structural mechanism for maintaining the ‘on-state’ of the CaMKII memory switch in the post-synaptic density.. J Neurochem.

[80] Vayttaden SJ, Bhalla US (2004). Developing complex signaling models using GENESIS/Kinetikit.. Sci STKE.

[81] Hoops S, Sahle S, Gauges R, Lee C, Pahle J (2006). COPASI—a COmplex PAthway SImulator.. Bioinformatics.

[82] Funahashi A, Tanimura N, Morohashi M, Kitano H (2003). CellDesigner: a process diagram editor for gene-regulatory and biochemical networks.. BIOSILICO.

