# Supplementary material for: Signaling Logic of Activity-Triggered Dendritic Protein Synthesis: An mTOR Gate But Not a Feedback Switch
Source: PLoS Comput Biol. 2009 Feb 13;5(2):e1000287. doi: 10.1371/journal.pcbi.1000287 (PMC2647780; doi:10.1371/journal.pcbi.1000287)
Supplement: Figure S1 — Comparison of responses of alternate model (model with MAPK activated PDK1 activity) with the reference model. Filled squares indicate responses from reference model and open triangles indicate responses from alternate model. Responses of : (A) PDK1, (B) PDK1*, (C) active AKT (PIP3_AKT_thr-308), (D) S6K_tot (sum total of the phosphorylated forms of S6K), (E) 40S (phosphorylated form of 40S), and (F) protein synthesis rate, after a delivery of steady stimulus of 3.7 nM BDNF. (0.09 MB PDF) [file pcbi.1000287.s004.pdf]

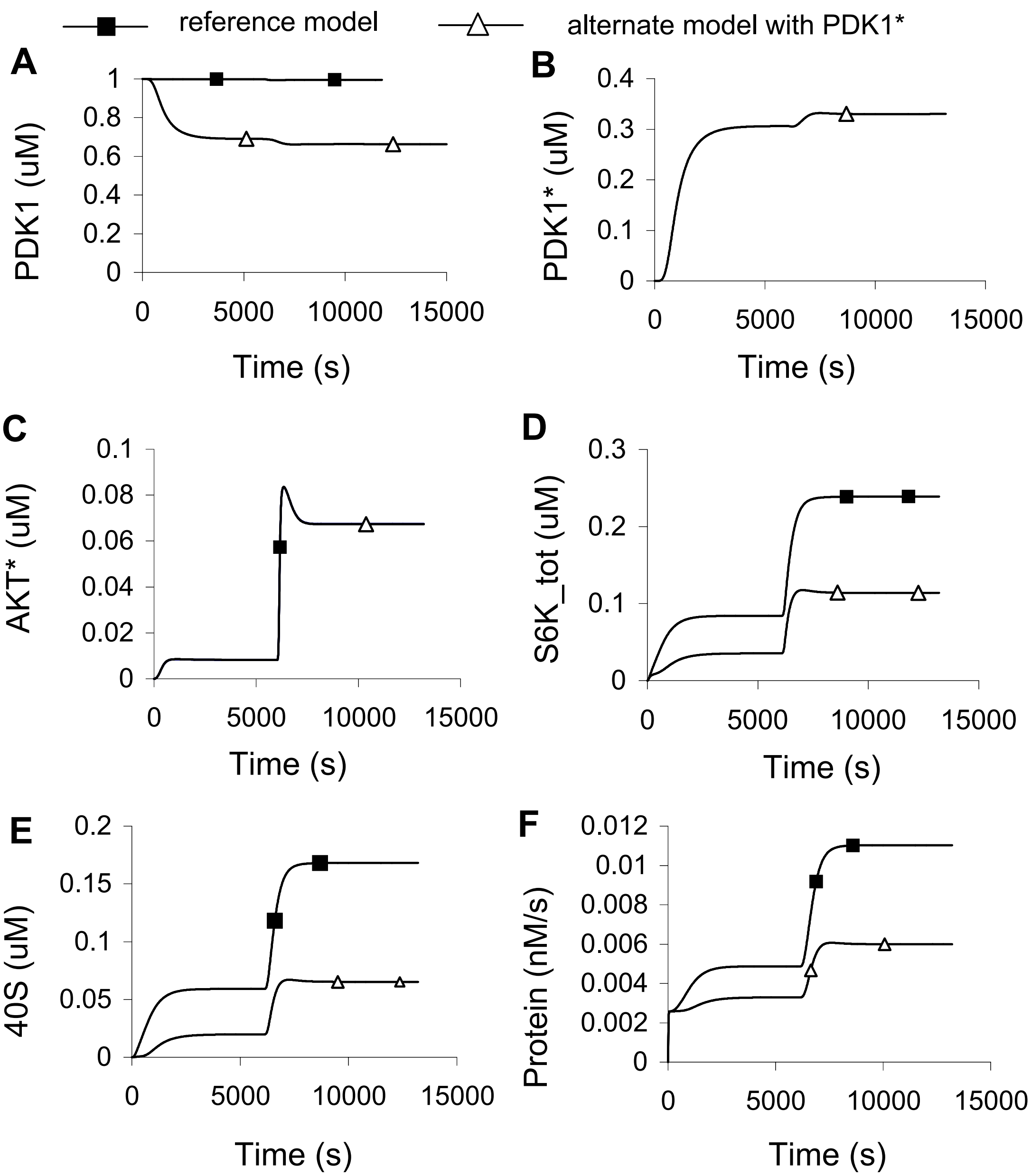

Supplementary Figure S1:
Comparison of responses of alternate model (model with MAPK activated PDK1 activity) with the reference model.
Filled squares indicate responses from reference model and open triangles indicate responses from alternate model.
Responses of :
A: PDK1, B: PDK1*, C: active AKT (PIP3_AKT_thr-308),
D: S6K_tot (sum total of the phosphorylated forms of S6K), E: 40S (phosphorylated form of 40S) and F: protein synthesis rate, after a delivery of steady stimulus of 3.7 nM BDNF.
