# Supplementary material for: Signaling Logic of Activity-Triggered Dendritic Protein Synthesis: An mTOR Gate But Not a Feedback Switch
Source: PLoS Comput Biol. 2009 Feb 13;5(2):e1000287. doi: 10.1371/journal.pcbi.1000287 (PMC2647780; doi:10.1371/journal.pcbi.1000287)
Supplement: Figure S2 — Diagram of existed signaling models inputs to the current model (A) Chemical reaction diagram of published model of PKC regulation showing its activation (B) Chemical reaction diagram of published model of MAPK pathway. (3.90 MB PDF) [file pcbi.1000287.s005.pdf]

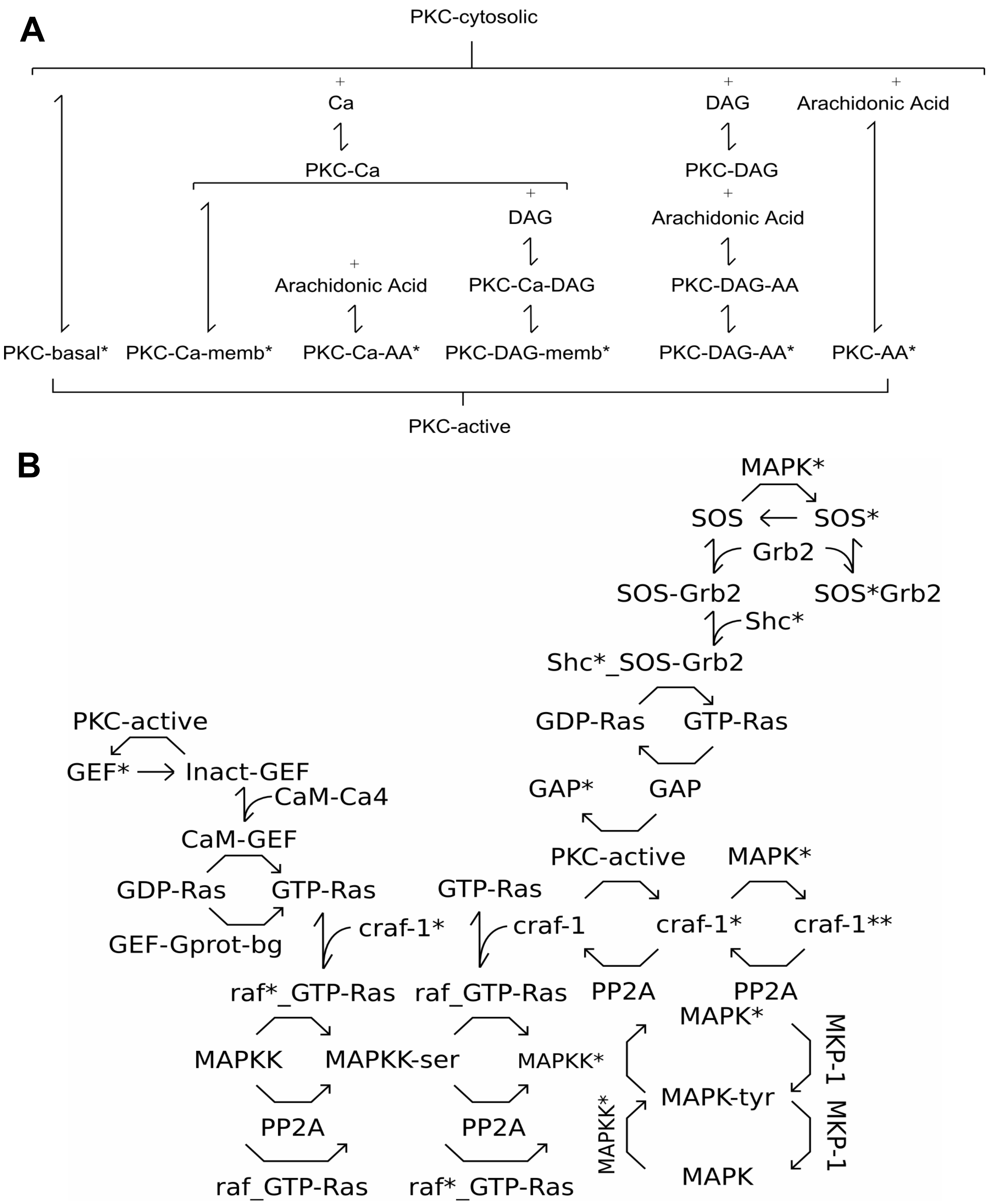

Supplementary Figure S2:

Diagram of existed signaling models inputs to the current model

A. Chemical reaction diagram of published model of PKC regulation showing its activation

B. Chemical reaction diagram of published model of MAPK pathway.
