# Supplementary material for: Signaling Logic of Activity-Triggered Dendritic Protein Synthesis: An mTOR Gate But Not a Feedback Switch
Source: PLoS Comput Biol. 2009 Feb 13;5(2):e1000287. doi: 10.1371/journal.pcbi.1000287 (PMC2647780; doi:10.1371/journal.pcbi.1000287)
Supplement: Figure S3 — Parameter sensitivity analysis. We systematically varied the total concentrations of each molecule (CoInit) from 0.1 to 10 fold the original model value. We plotted active AKT, MAPK*, S6K_tot (sum total of the phosphorylated forms of S6K) and 4E-BP_tot (sum total of the phosphorylated forms of 4E-BP). (0.02 MB PDF) [file pcbi.1000287.s006.pdf]

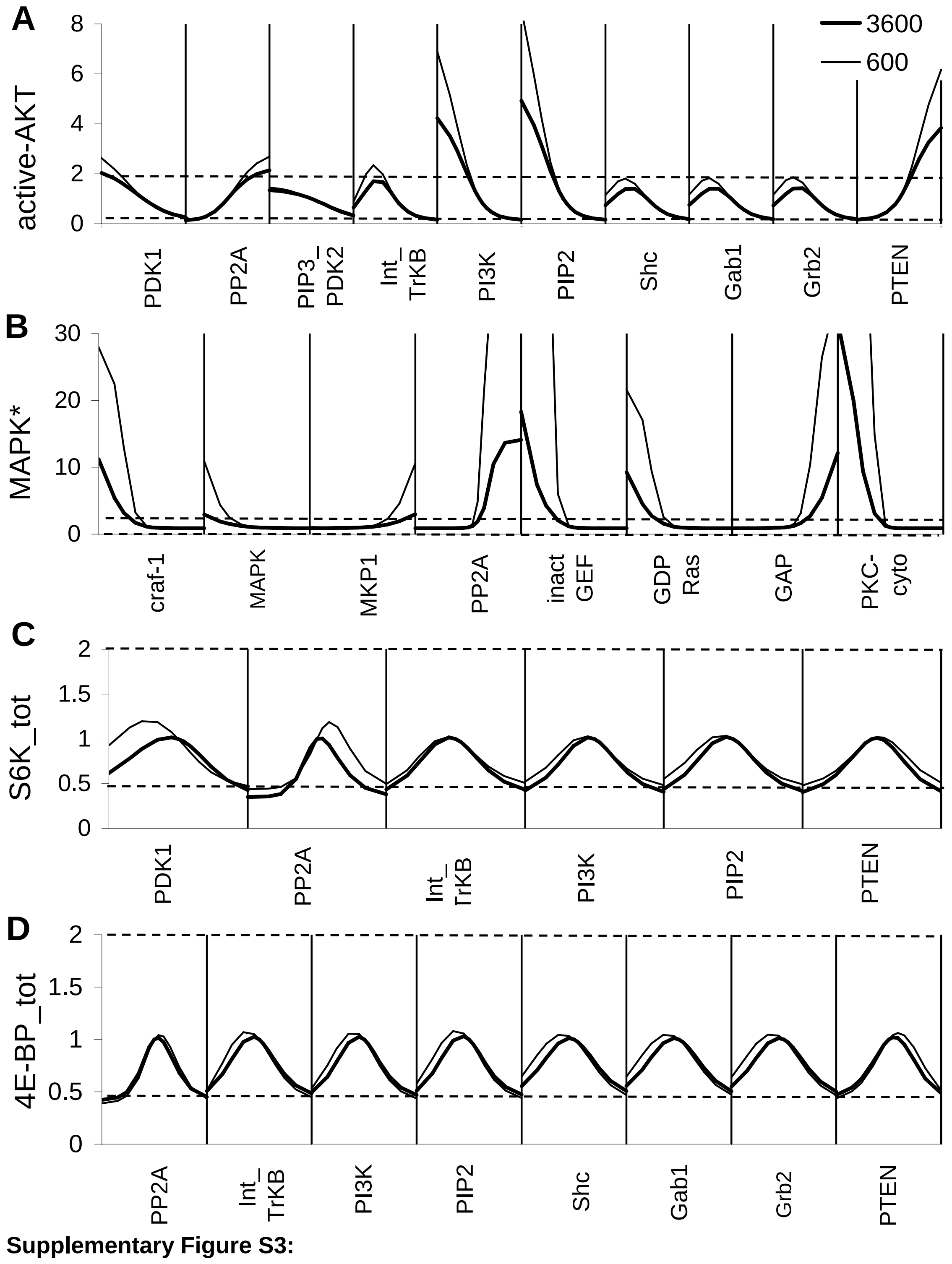


**Supplementary Figure S3:**

Parameter sensitivity analysis. We systematically varied the total concentrations of each molecule (CoInit) from 0.1 to 10 fold the original model value. We plotted active AKT, MAPK*, S6K_tot (sum total of the phosphorylated forms of S6K) and 4E-BP_tot (sum total of the phosphorylated forms of 4E-BP).
