# Supplementary material for: Signaling Logic of Activity-Triggered Dendritic Protein Synthesis: An mTOR Gate But Not a Feedback Switch
Source: PLoS Comput Biol. 2009 Feb 13;5(2):e1000287. doi: 10.1371/journal.pcbi.1000287 (PMC2647780; doi:10.1371/journal.pcbi.1000287)
Supplement: Figure S5 — Parameter sensitivity analysis. We systematically varied the kcat of each molecule from 0.1 to 10 fold the original model value. (0.01 MB PDF) [file pcbi.1000287.s008.pdf]

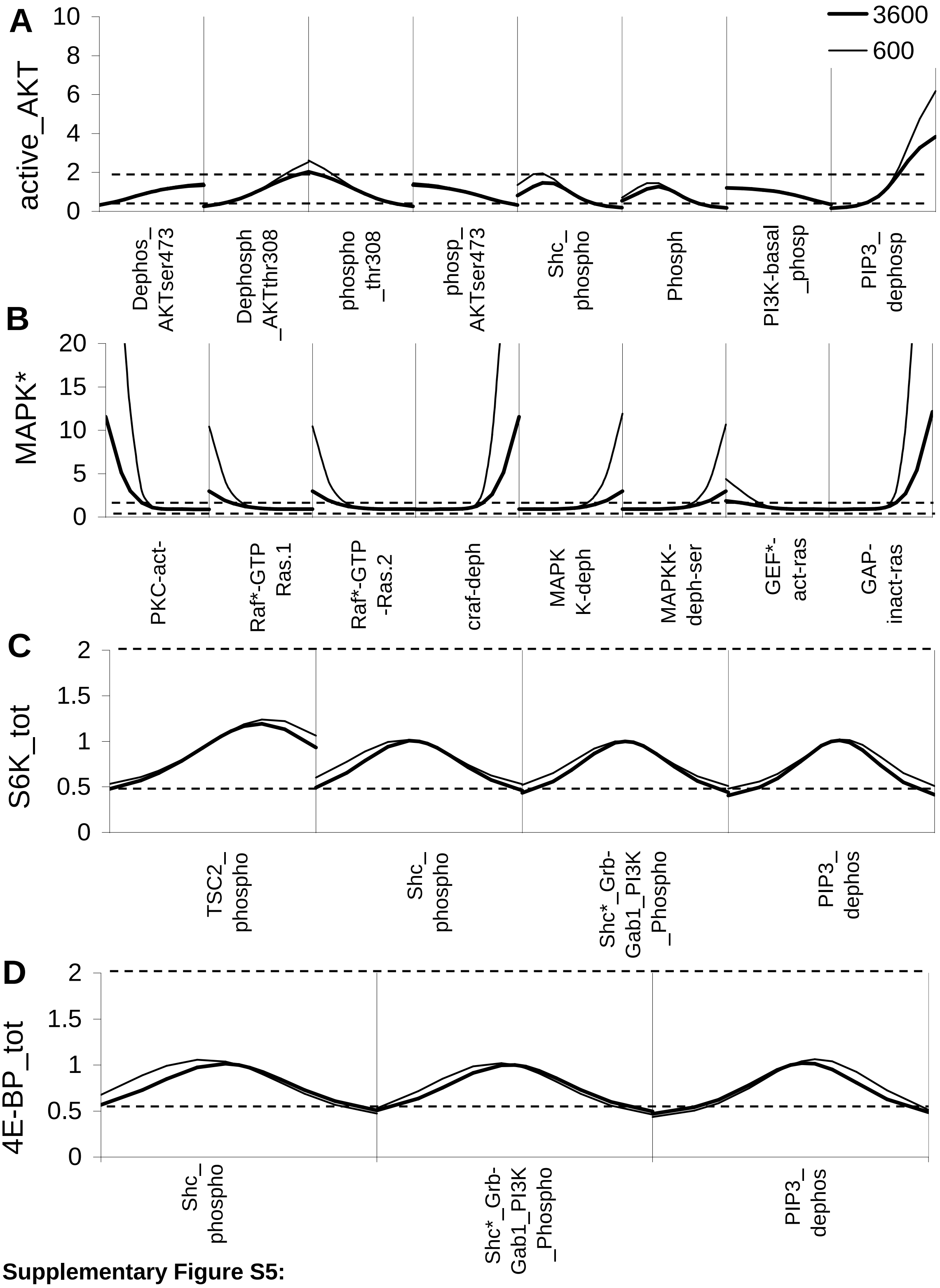

Supplementary Figure S5:

Parameter sensitivity analysis. We systematically varied the kcat of each molecule from 0.1 to 10 fold the original model value.
