# Supplementary material for: Signaling Logic of Activity-Triggered Dendritic Protein Synthesis: An mTOR Gate But Not a Feedback Switch
Source: PLoS Comput Biol. 2009 Feb 13;5(2):e1000287. doi: 10.1371/journal.pcbi.1000287 (PMC2647780; doi:10.1371/journal.pcbi.1000287)
Supplement: Figure S6 — Parameter sensitivity analysis. We systematically varied the Kf of each molecule. (0.01 MB PDF) [file pcbi.1000287.s009.pdf]

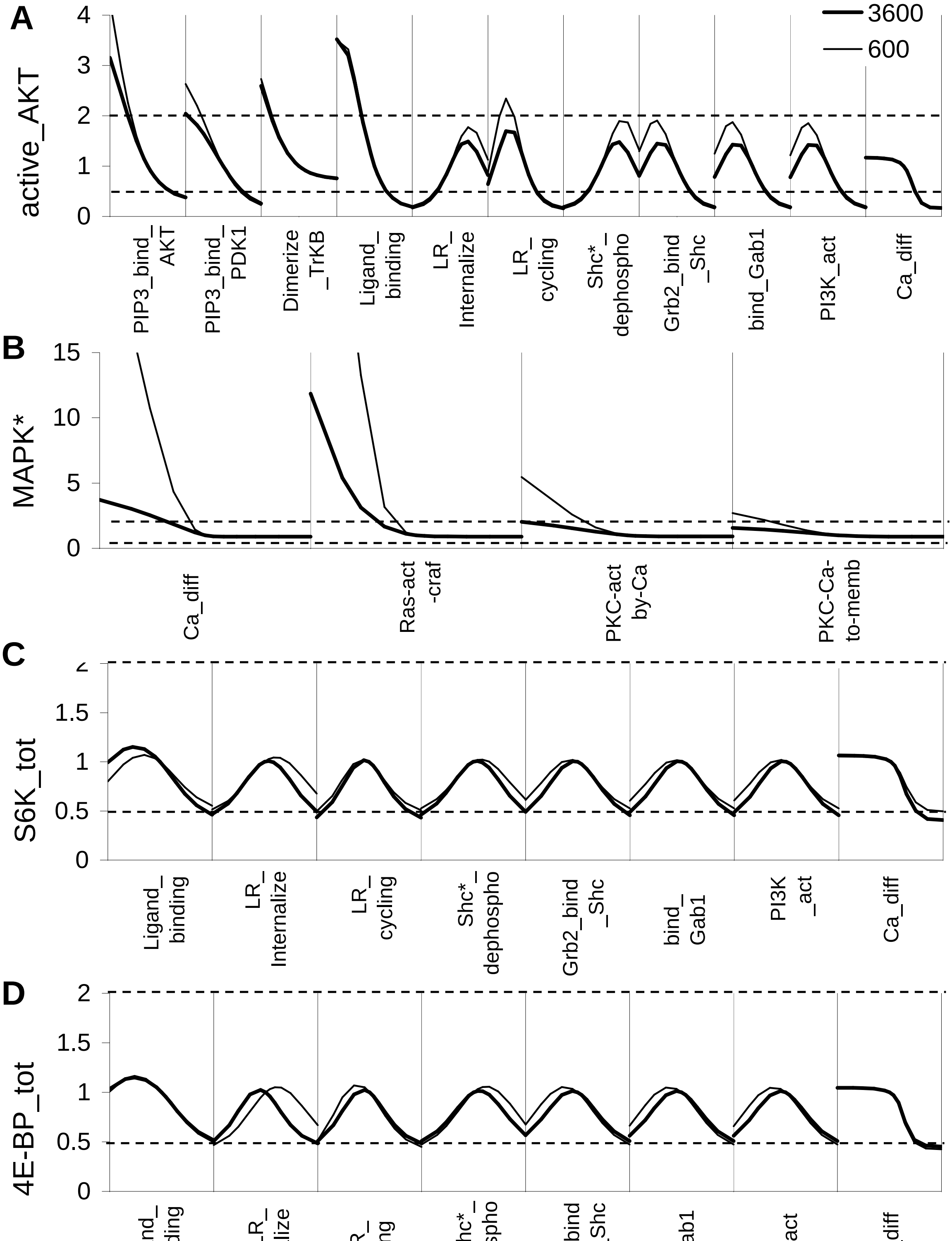

Supplementary Figure S6:

Parameter sensitivity analysis. We systematically varied the Kf of each molecule.
