# Supplementary material for: Signaling Logic of Activity-Triggered Dendritic Protein Synthesis: An mTOR Gate But Not a Feedback Switch
Source: PLoS Comput Biol. 2009 Feb 13;5(2):e1000287. doi: 10.1371/journal.pcbi.1000287 (PMC2647780; doi:10.1371/journal.pcbi.1000287)
Supplement: Figure S8 — Dose response of protein synthesis rate as a function of active MAPK. BDNF is buffered at basal level (0.05 µM (A)) and at stimulated level (3.7 nM (B)).The basal kinase activity is 0.01 /sec. There is a weak dependence of protein synthesis rate on MAPK activity. (0.08 MB PDF) [file pcbi.1000287.s011.pdf]

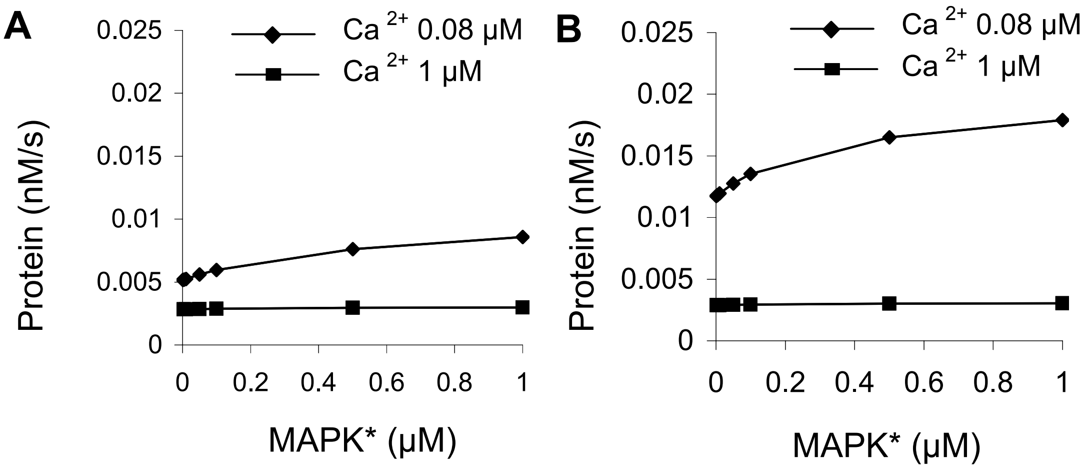

Supplementary Figure S8:
Dose response of protein synthesis rate as a function of active MAPK. BDNF is buffered at basal level (0.05 µM Figure S8 A) and at stimulated level (3.7 nM, Figure S8 B).The basal kinase activity is 0.01 /sec. There is a weak dependence of protein synthesis rate on MAPK activity.
