# Supplementary material for: Signaling Logic of Activity-Triggered Dendritic Protein Synthesis: An mTOR Gate But Not a Feedback Switch
Source: PLoS Comput Biol. 2009 Feb 13;5(2):e1000287. doi: 10.1371/journal.pcbi.1000287 (PMC2647780; doi:10.1371/journal.pcbi.1000287)
Supplement: Figure S9 — Response to LTP and LTD stimuli. (A) protein synthesis for the LTP stimuli and (B) protein synthesis for the LTD stimuli. LTP stimulus was 3 Ca2+ peaks of 10 µM for 1 sec each, separated by 5 min (Arrows below time axis in (A)). Filled triangles indicate runs where the Ca2+ remained at baseline (0.08 µM) and open squares indicate Ca2+ at 10 µM. The LTD stimulus (Bar below time axis in (B)) was a single pulse of Ca2+ for 900 sec at 1 µM (open squares) and 0.2 µM Ca2+ (filled triangles). The simulations are with zero BDNF. (0.09 MB PDF) [file pcbi.1000287.s012.pdf]

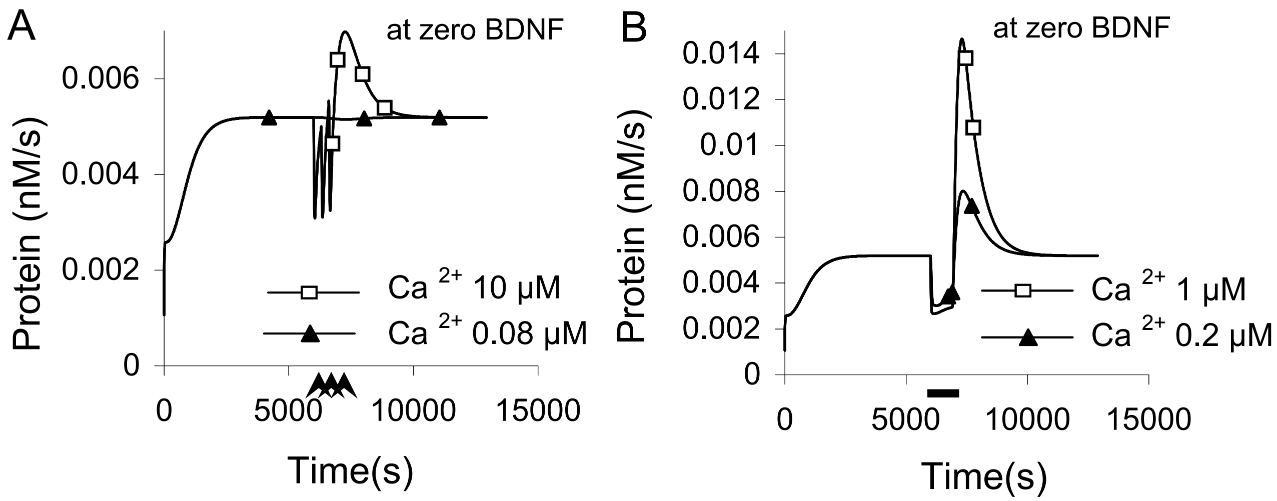

Supplementary Figure S9:

Response to LTP and LTD stimuli.

A: protein synthesis for the LTP stimuli and

B: protein synthesis for the LTD stimuli.

LTP stimulus was 3 Ca^{2+} peaks of 10 μM for 1 sec each, separated by 5 min (Arrows below time axis in A). Filled triangles indicate runs where the Ca^{2+} remained at baseline (0.08 μM) and open squares indicate Ca^{2+} at 10 μM.

The LTD stimulus (Bar below time axis in B) was a single pulse of Ca^{2+} for 900 sec at 1 μM (open squares) and 0.2 μM Ca^{2+} (filled triangles).

The simulations are with zero BDNF.
